# Unsupervised clustering reveals noncanonical myeloid cell subsets in the brain tumor microenvironment

**DOI:** 10.1007/s00262-024-03920-1

**Published:** 2025-01-03

**Authors:** Ismaïl Hermelo, Tuomo Virtanen, Iida Salonen, Reetta Nätkin, Sofia Keitaanniemi, Aliisa M. Tiihonen, Suvi Lehtipuro, Laura Kummola, Ella Raulamo, Kristiina Nordfors, Hannu Haapasalo, Minna Rauhala, Juha Kesseli, Matti Nykter, Joonas Haapasalo, Kirsi Rautajoki

**Affiliations:** 1 Prostate Cancer Research Center, Faculty of Medicine and Health Technology, Tampere University and Tampere University Hospital, Tampere, Finland; 2 Unit of Pediatric Haematology and Oncology, Tampere University Hospital, Tampere, Finland; 3 Tays Cancer Center, Tampere University Hospital and Tampere University, Tampere, Finland; 4 Faculty of Medicine and Health Technology, Tampere University, Tampere, Finland; 5 Fimlab Laboratories Ltd, Tampere University Hospital, Tampere, Finland; 6 Department of Neurosurgery, Tampere University Hospital, Tampere, Finland; 7 Tampere Institute for Advanced Study, Tampere University, Tampere, Finland

**Keywords:** Glioblastoma, Brain metastasis, Tumor microenvironment, Immunophenotyping, CD3^+^ myeloids, CD19^+^ myeloids, Unsupervised clustering, Single-cell sequencing, Deconvolution

## Abstract

The tumor immune microenvironment (TiME) of human central nervous system (CNS) tumors remains to be comprehensively deciphered. Here, we employed flow cytometry and RNA sequencing analysis for a deep data-driven dissection of a diverse TiME and to uncover noncanonical immune cell types in human CNS tumors by using seven tumors from five patients. Myeloid subsets comprised classical microglia, monocyte-derived macrophages, neutrophils, and two noncanonical myeloid subsets: CD3^+^ myeloids and CD19^+^ myeloids. T lymphocyte subsets included double-negative (CD4^−^ CD8^−^) T cells (DNTs). Noncanonical myeloids and DNTs were explored on independent datasets, suggesting that our DNT phenotype represents *γδ* T cells. Noncanonical myeloids were validated using orthogonal methods across 73 patients from three independent datasets. While the proportions of classical myeloids agreed with reported malignancy type-associated TiMEs, unexpectedly high lymphocyte frequencies were detected in gliosarcoma, which also showed a unique expression pattern of immune-related genes. Our findings highlight the potential of data-driven approaches in resolving CNS TiME to reveal the mosaic of immune cell types constituting TiME, warranting the need for future studies on the nonclassical immune cell subsets.

**Supplementary Information:**

The online version contains supplementary material available at 10.1007/s00262-024-03920-1.

## Introduction

Tumors of the CNS can generally be categorized as CNS-native, including diffuse gliomas, or CNS-invading, such as brain metastases (BrMs) [1, 2]. Diffuse gliomas are classified based on the presence of *isocitrate dehydrogenase* (*IDH*) *1* and *2* hotspot mutations [3]. Primary *IDH*-mutant gliomas (IDH^mut^) are generally low grade (2 or 3) and are associated with a better prognosis (− 57 months) than *IDH* wild-type (IDH^wt^) grade 4 glioblastomas (GB) (− 14 months) [4]. BrMs are typically lung, melanoma or breast carcinomas and are associated with a poor prognosis (7, 7 and 9 months, respectively) [5, 6].

The CNS TiME is immunosuppressive. CNS-resident (microglia [MG]) [5] and CNS-infiltrating (monocyte-derived macrophages [MDMs]) [6] myeloid cells are a prominent feature in diffuse glioma TiME, accounting for up to 50% of the cells in the tumor mass [5–8]. It has also been shown that MDM and MG (i.e., tumor-associated microglia/macrophages, TAMs) states and accumulations can differ between newly diagnosed or recurrent gliomas [9]. Moreover, CNS TiME also includes tumor-associated neutrophils (TANs) with various inflammatory phenotypes [7, 8, 10], which accumulate more profoundly in IDH^wt^ gliomas and BrMs than in IDH^mut^ gliomas [10].

In addition, lymphoid lineage-derived immune cells are also part of the CNS TiME, including adaptive lymphoid infiltrates such as cytotoxic (CD8^+^), helper (CD4^+^), and regulatory (FoxP3^+^) T cells. The latter is reported to preferentially accumulate in BrMs when compared to gliomas [5–8]. Regulatory T cells secrete soluble mediators (e.g., interleukin [IL]-4, IL-10 and IL-13), thus contributing to CNS TiME-suppressive properties and resulting in the repression of cytotoxic CD8 T-cell responses with exhausted features [7]. Moreover, innate lymphoid infiltrates also comprise the CNS TiME lymphoid compartment, such as NK cells (CD56^+^) and *γδ* T cells (*γδ* TCR^+^) [7]. Similar to regulatory T cells, the TiME dictates whether *γδ* T cells exhibit inflammatory or suppressive roles [7, 8, 11].

Nonetheless, single-cell measurements pose challenges in the identification and categorization of human immune cell phenotypes, especially disease-associated phenotypes, different from steady-state phenotypes (traditionally from peripheral blood analysis) and thus likely yet to be fully described. TAM subsets illustrate that definitions are currently lacking, while certain marker proteins are used for their separation, such as CCR2 for MDMs and TMEM119 or P2RY12 for MGs [7–9]. Furthermore, both MDMs and MGs can be subgrouped based on gene signatures and marker combinations [9]. However, nonclassical monocytes were also detected in CNS tumors [8, 9], therefore revealing unknown CNS TiME constituents.

Together, these studies report frequencies and signature states of MDMs, TANs or regulatory T cells, which have been shown to accrue in IDH^wt^ gliomas and BrMs, while IDH^mut^ gliomas display larger MG frequencies and fewer T lymphocytes [7–9, 12]. However, these studies centered around landmark immune cell types and categorized them based on predefined marker combinations considered for TiME analysis (e.g., flow cytometry [FCM]) [8] or, if using automated cell subpopulation identification methods (e.g., clustering analysis), expert-guided manual metaclustering [7].

Accordingly, we aimed to address human brain tumor immune cells without deterministic cell surface marker groups by leveraging an unsupervised computational approach. Therefore, in this study, we investigated whether the brain TiME could include immune subsets beyond classical cell types across CNS tumor malignancies.

## Material and methods

### Reagents

Tumor dissociation kit TDK (Miltenyi Biotec) 130–095-929.

Debris removal solution (Miltenyi Biotec) 130–109-398.

CD45 Microbeads (Miltenyi Biotec) 130–045-801.

MS columns (Miltenyi Biotec) 130–042-201.

miniMACS separator (Miltenyi Biotec) 130–042-102.

Human Fc Block, (BD Bioscience) Cat#564,220.

Fixable Viability Stain 510, (BD Bioscience) Cat#564,406.

CD4 APC-H7 mouse monoclonal anti-human, (clone RPA-T4), (BD Bioscience) Cat#564,406.

CD3 BV421 mouse monoclonal anti-human, (UCHT1), (BD Bioscience) Cat#562,426.

CD8 BV650 mouse monoclonal anti-human, (RPA-T8), (BD Bioscience) Cat#563,821.

CD66b AF647 mouse monoclonal anti-human, (G10F5), (BD Bioscience) Cat#561,645.

CD14 AF700 mouse monoclonal anti-human, (G10F5), (BD Bioscience) Cat#557,923.

CD19 AF488 mouse monoclonal anti-human, (G10F5), (BD Bioscience) Cat#557,697.

CD45 BV786 mouse monoclonal anti-human, (HI30), (BD Bioscience) Cat#563,716.

CompBeads Antimouse Igk/Negative control, (BD Bioscience) Cat#552,843.

### In-house cohort

Tumor samples analyzed with FCM (five patients, seven samples) and/or transcriptome bulk-RNA sequencing (RNA-seq) (three patients, five samples) were collected for this study from patients operated at Tampere University Hospital. Experienced neuropathologist evaluated the tumor specimens and determined the histopathological type and grade, according to the criteria presented by the World Health Organization (WHO) 2016.

### FCM analysis

Tumor tissue samples were immediately dissociated into single-cell suspensions after surgical resection. After mechanical dissociation with scalpels, enzymatic digestion with tumor dissociation kit (Miltenyi) was performed, followed by gradient centrifugation using debris removal solution (Miltenyi) according to the manufacturer’s protocols.

CD45-positive immune cells were sorted using magnetic cell sorting following manufacturer’s recommendations (CD45 Microbeads, MS columns and miniMACS separator from Miltenyi). Single-cell suspensions were used for FCM analysis and RNA-seq.

For FCM analysis, cells were stained with Fixable Viability dye (BD Biosciences) for 6 min in 37 °C at the dark, followed by 10 min RT incubation with Fc receptor blocking solution (5 µL/million cells, BD Biosciences). Next, antibody staining mix was added and incubated for 20 min at 4 °C protected from light. After washing cells with FCM buffer (phosphate-buffered saline [PBS], 0.5% bovine serum albumin [BSA], 2 mM ethylenediaminetetraacetic acid [EDTA]) samples were acquired using flow cytometer (BD Aria Fusion, Aria III flow cytometer).

### Phenocluster generation

**Fig. 1:**
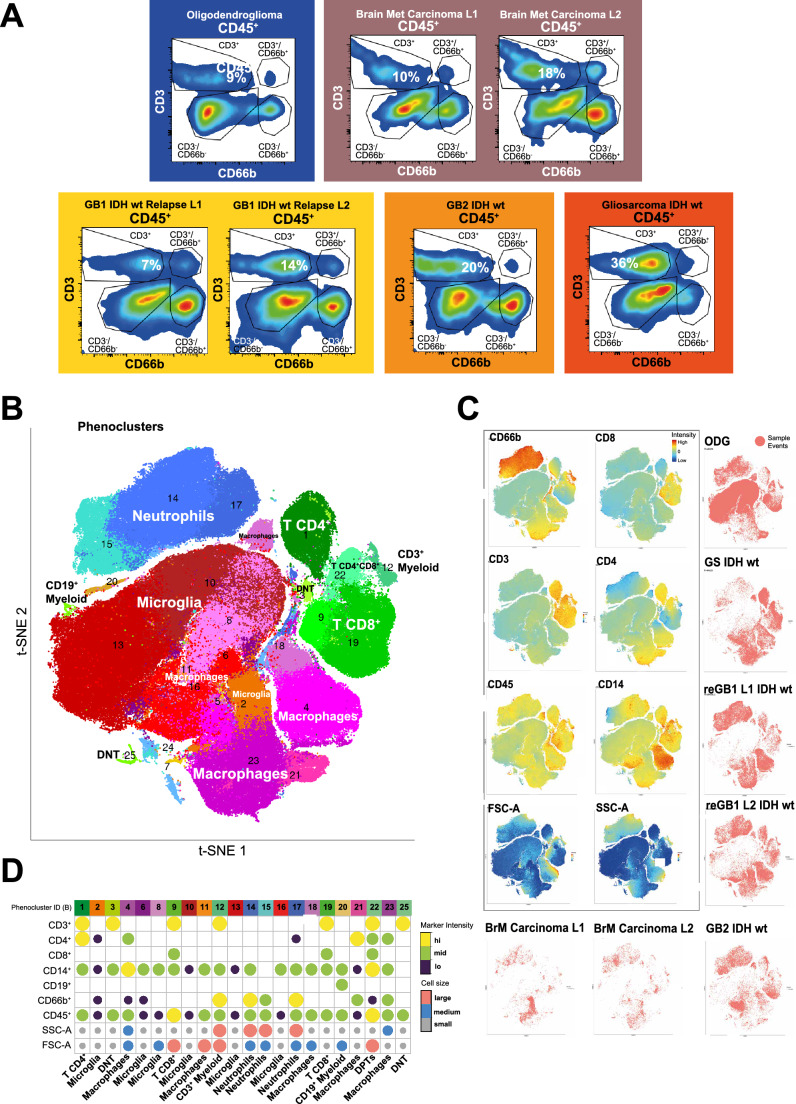
Flow cytometry analysis of CNS tumors reveals classical and noncanonical cell phenotypes. Unsupervised clustering identifies further myeloid population heterogeneity in brain cancers, identifying subsets of noncanonical myeloids: CD19^+^ myeloid cells and CD3^+^ myeloid cells. **A** Sequential bivariate density plots outline general immune population strata in viable CD45 + cells. Samples can be sorted from CD45^+^CD3^+^ low (oligodendroglioma) to high (gliosarcoma). Noncanonical CD3^+^CD66b^+^ cells were also detected in most samples. **B** Unsupervised PhenoGraph clustering and t-SNE visualization of viable CD45^+^ cells organized 259,636 cells from seven tumor samples into 25 clusters (phenoclusters) and 10 cell types. Cells are colored by phenocluster ID (in Panel D), and the representative cell types are written on each cluster; t-SNE, t-distributed stochastic neighbor embedding. **C** tSNE visualizations of fluorescent intensities for the indicated markers, cell size (FSC-A) and granularity (SSC-A (left)). Samplewise cell distributions within viable CD45^+^ events t-SNE (right, bottom); FSC-A, forward scatter area; SSC-A, side scatter area; ODG, oligodendroglioma; reGBM, recurrent glioblastoma; GBM, primary glioblastoma; GS, gliosarcoma; BrM, brain metastasis; L1, tumor locus 1; L2, tumor locus 2. **D** Plot of cell marker expression levels (lo, mid, hi) and forward scatter area (size) of phenoclusters (color scheme as in B), including phenotype annotations

Doublets and dead cells were removed by manual gating using FlowJo v10 software. The data was imported to* R* (version 3.6.0) and it was transformed using autoLgcl-function from cytofkit package (version 1.4.4), which estimates the parameters for logicle-transformation automatically. To ensure that unstained control samples underwent the same transformation, the matched stained and unstained samples were merged and transformed together. All the stained events across samples were merged and clustered using PhenoGraph [13] clustering algorithm with k-value of 60 (https://github.com/JinmiaoChenLab/Rphenograph). Clustering was performed in two rounds. After the first round, clusters with low CD45 signal were filtered out to enrich for CD45^+^ events which were clustered again and visualized with t-distributed stochastic neighbor embedding projection, using Rtsne function in* R* (version 0.15). In house-FCM data was normalized using FlowJo v10 software (Fig. 1A); similar analysis performed on FCM data from [7].

Pearson correlation between samples or clusters was calculated from the fractions of events belonging to each phenocluster per sample (or cell type, in the cell types correlation analysis). Pearson correlation p-values were calculated using cor.test function in *R* (version 3.6.0).

### RNA-seq data processing.

Raw sequencing reads from RNA-seq experiments of 156 primary GBM samples generated by The Cancer Genome Atlas (TCGA) (Cancer Genome Atlas Research Network, 2008) were downloaded from the NCI Genomic Data Commons. The read alignment and read count quantification of in-house RNA-seq samples and TCGA samples were performed with STAR version 2.5.3a [14], using Ensembl reference genome GRC38 and Gencode annotation release 29. Raw RNA-sequencing read counts of samples representing six different cell types/groups (CD45^−^, MG, MDM, neutrophils, CD4^+^ T cells and CD8^+^ T cells), purified with flow cytometry sorting, were acquired form [8] and used as cell type references. Only samples from patients with histology annotated as “Glioblastoma” were included into this reference set. Read counts in all the samples were TPM and quantile-normalized (R-package preprocessCore version 1.62.1) and log2-transformed for subsequent analysis.

### Deconvolution of in-house and TCGA RNA-seq samples

Cell type reference set included 16 samples for CD45^−^ cell type, 14 samples for MG cell type, 12 samples for MDM cell type, 11 samples for neutrophils, 10 samples for CD4^+^ T cells and 5 samples for CD8^+^ T cells. Median expression values of the samples for each cell type were used for calculating the cell type references. Cell type compositions of the in-house and TCGA RNA-seq samples were deconvolved with linear regression analysis as previously described [15]. No median sample was used, and genes used in deconvolution were limited to genes found in previously described immune-related gene clusters [15]. Linear regression with elastic-net regularization was performed with R-package glmnet version 4.1–7, and elastic-net mixing parameter alpha was set to 0.25. The regularization parameter lambda was chosen using tenfold cross-validation. R-package pheatmap version 1.0.12 was used to visualize the deconvolution results.

### Identification of candidate fusion transcripts from RNA-sequencing of in-house samples

RNA-sequencing reads were aligned with STAR version 2.7.2a [14] allowing chimeric alignment using the following parameters:

“--outSAMtype BAM SortedByCoordinate --outReadsUnmapped None --twopassMode Basic --readFilesCommand "gunzip -c" --outSAMstrandField intronMotif --outSAMunmapped Within --chimSegmentMin 12 --chimJunctionOverhangMin 8 --chimOutJunctionFormat 1 --alignSJDBoverhangMin 10 --alignMatesGapMax 100000 --alignIntronMax 100000 --alignSJstitchMismatchNmax 5 -1 5 5 --outSAMattrRGline ID:GRPundef --chimMultimapScoreRange 3 --chimScoreJunctionNonGTAG -4 --chimMultimapNmax 20 --chimNonchimScoreDropMin 10 --peOverlapNbasesMin 12 --peOverlapMMp 0.1 --alignInsertionFlush Right --alignSplicedMateMapLminOverLmate 0 --alignSplicedMateMapLmin 30”

Human genome and annotations provided in Trinity Cancer Transcriptome Analysis Toolkit (CTAT) genome lib (GRCh38 v37 Mar012021) were used in alignment. STAR-Fusion version 1.10.0 [16] with provided singularity image and aforementioned CTAT genome lib was used to identify the candidate fusion transcripts from in-house samples.

### Immune cluster activities on in-house and TCGA RNA-seq samples

Cluster activities for previously described immune-related gene clusters [15] were calculated and scaled from 0 to 1 as described before in [15]. Genes with low variance (< 0.05) across the samples were filtered out from the analysis. Cluster activity was calculated as a median of the largest group of genes in the cluster having a positive pairwise correlation with each other. Before taking the median, expression values of each gene were scaled from 0 to 1 with R-package scales version 1.3.0 after the values below the second and above the 98th percentiles were assigned to 0 and 1, respectively, to remove outliers from the data. R-package pheatmap version 1.0.12 was used to visualize the immune cluster activities in in-house samples.

### Expression analysis of a previously published immune-gene set in in-house samples

The expression of the genes from an immune-gene set described in [17] was inspected on in-house samples. Their median-corrected expression values were visualized using R-package pheatmap version 1.0.12.

### Correlations of cell type composition, immune cluster activity and immune-gene set expressions

Pearson and Spearman correlations across samples were calculated for deconvolution coefficient values, cluster activities and between them. Only Spearman correlations are shown. The correlation results were visualized with R-package corrplot version 0.92.

### CyTOF data processing

Mass cytometry data from [7] were downloaded from Mendeley Data repository, normalized using Cytofkit arcsinh function and clustered using RPhenograph included in Cytofkit package (version 0.99.0) (k = 60). Due to practical reasons, the myeloid panel of the dataset comprising 26 patient samples was filtered to contain only CD45^+^ cells (CD45 expression > 0.5 after normalization) and a maximum of 500,000 cells per sample, resulting in altogether 9,778,719 CD45^+^ cells. Only a selected subset of markers was used for cell clustering (namely CD45, CD19, CD206, CD123, CD64, CD11c, CX3CR1, CD66b, CD45RA, CD1c, CD86, CD141, CD169, CD14, CD163, CD16, CD209, CD3, CD68, CD88, CD56, CD11b and CCR2). As the 18 patient samples in the lymphoid panel did not contain as many cells (1,700,546 cells in total), lymphoid panel data were not filtered. Visualizations were obtained using uniform manifold approximation and projection (UMAP) dimension reduction.

Cell types were annotated manually based on the marker combinations. After cell-type annotations, the clusters containing cells with high viability score (i.e., non-viable cells) and cells not presenting immune cell characteristics, labeled as “Other cells”, were removed from the dataset. Then, the dimensionality reduction UMAP was performed using the filtered dataset. Proportions of cell types were calculated against all the analyzed CD45^+^ cells in the myeloid panel (max 500 000 cells per sample), and for the cells in the lymphoid panel against were calculated all cells (i.e., no CD45^+^ distinction). The correlation between cell-type proportions across samples was calculated using Pearson correlation.

### Single-cell RNA-seq (SCS) data analysis

Data were downloaded from [9, 18–20]. Data were preprocessed in *R* (version 4.1.2) to contain cells with expression from minimum 200 genes, and genes were included if expressed in at least 3 cells. The mitochondrial gene expression percentage in each cell had to be under 5% to be included. After this, the copy number alterations were inferred using the cell expression medians visualized with InferCNV (version 1.10.1) with window length 301 (https://github.com/broadinstitute/inferCNV). Cells were organized into 5 (Slyper, Richards) or 10 (Neftel) clusters depending on the dataset, and copy number altered clusters were identified based on the presence of chromosome 7 gain and 10 loss as well as other characteristic alterations.

Data were integrated using the Seurat Wrapper [21] for Harmony [22] (version 1.2.0) with 2000 variable features, 30 principal components and original identities as the grouping variable. The copy number variation status (InferCNV) was used to remove tumor cells from the data, and the rest of the data was converted into a Seurat (version 4.3.0) object. These non-CNV altered cells were then clustered using resolution 2.5 with Seurat’s FindClusters-function. After this, phenotype identities were manually assigned for each cluster based on the marker gene expression.

For the Luoto et al.’s immune cluster (ICs) gene analysis in SCS data, average expression in each cell type was calculated for each gene within IC gene clusters using Seurat AverageExpression. To point in which cells IC genes were overexpressed, we used mean + 0.5 STD as threshold for overexpression for each gene. Then, genes in each IC were divided into subclusters using* R* hclust based on the overexpression status as a binary variable (Supplementary Table 3). For each of these subclusters, the fraction of overexpressed genes was calculated for each cell type and visualized as dotplots.

Differential expression (DE)-gene analysis was performed for CD3^+^ myeloid-identified cells versus selected cell types or all other CD45^+^ cells in the dataset using the MAST wrapper for Seurat (version 1.20.0). For a gene to be considered a DE-gene, the logarithmic fold change had to be over 0.25 and the Bonferroni-adjusted p-value had to be under 0.05. For the over-representation analysis (ORA), we used clusterProfiler [23] (version 4.2.2), with adjusted p-value cutoff of 0.05. The ORA was run separately for downregulated and upregulated DE genes.

### Statistical analysis

Statistical testing was performed using* R* (version 4.1.2). Statistical tests used are described in appropriate sections within methods. Indicated p-values were calculated using two-sided Wilcoxon test.

### Multiplex immunohistochemistry validation cohort

Formalin-fixed paraffin-embedded (FFPE) whole mount tumor sample blocks from 14 diffuse glioma samples from 9 patients were used as independent validation cohort. An experienced neuropathologist evaluated the FFPE tumor samples and determined the histopathological type and grade according to the criteria presented by the World Health Organization (WHO).

### Multiplex immunohistochemical staining

Tumor samples were fixed in 4% phosphate-buffered formaldehyde and processed into paraffin blocks. Four-micrometer-thick sections were cut and stained with in-house multiplex immunohistochemistry (mIHC) protocol originally based on Multiple Iterative Labeling by Antibody Neodeposition by [24]. Antigen retrieval was done using a Tris–HCl buffer (pH 9.0) prior to antibody labeling in 121℃ for 20 min. Non-specific epitopes were blocked using normal goat serum (S-1000, Vector Laboratories) 1:20 for 5 min in room temperature, and autofluorescence was treated with 0,1% Sudan Black solution (199,664, Sigma-Aldrich) for 5 min in room temperature. Antigens were stained with anti-CD11c (D3V1E, Cell signaling technology, #45581S) 1:100, anti-CD68 (EPR20545, Abcam, #ab213363) 1:400, anti-CD45 (D9M8I, Cell signaling technology, #13917S) 1:200, anti-TMEM119 (polyclonal, Prestige Antibodies, #HPA051870) 1:500, anti-CD3ε (D7A6E, Cell Signaling Technology, #85,601) 1:200, anti-CD4 (N1UG0, Invitrogen, #14–2444-82) 1:50, and anti-CD8 (4B11, Leica Biosystems, #NCL-L-CD8-4B11) 1:10. Detection was done with Goat anti-Rabbit IgG (H + L) Highly Cross-Adsorbed Secondary Antibody, Alexa Fluor Plus 647 (Invitrogen, A32733) 1:500 and Goat anti-Mouse IgG (H + L) Cross-Adsorbed Secondary Antibody, Alexa Fluor 750 (Invitrogen, A21037) 1:50. Samples were mounted with Fluoromount-G (Invitrogen, 00–4959-52) mounting medium that contained DAPI for tissue counterstain. Stainings were scanned using a whole-slide scanner (NanoZoomer S60, Hamamatsu) using 387 nm, 650 nm and 740 nm excitation wavelengths.

### Multiplex-IHC image registration

The DAPI staining images were registered utilizing in-house scripts and Python package VALIS (version 1.0.0rc13)[25]**.** DAPI staining image of CD45 staining was used as the fixed reference. The transformation M-matrices obtained were applied to each matching staining image to obtain images in the same orientation. Regions of interest (ROI) were obtained by selecting 5000px x 5000px areas from the samples based on the tissue quality in H&E staining images. These obtained coordinates were transformed to original images to crop ROIs with the original resolution. ROI Images were processed into hyperstacks using Fiji-ImageJ (version 1.53v) [26] and analyzed manually in Qupath (version 0.4.4.).

## Results

### Flow cytometry analysis of CNS tumors reveals affluent immune population phenotypes, including classic and noncanonical cell phenotypes

We collected a heterogeneous cohort of seven samples from five freshly resected human CNS tumors to dissect diverse TiMEs and to uncover noncanonical immune cell types in CNS tumors. The cohort comprised glioma samples (1 IDH1^mut^ oligodendroglioma, 1 primary IDH1^wt^ glioblastoma, 1 primary IDH1^wt^ gliosarcoma, 2 recurrent IDH1^wt^ glioblastoma from same individual) and 2 breast cancer metastatic samples from the same patient (Table 1).

**Table 1:** Patient samples on in-house flow cytometry cohort

Sample ID	Gender	Age range	Primary or recurrence	Diagnostic	WHO grade*	Additional molecular features	Treatment prior to surgery
reGB1 L1, L2	M	(65–69)	3^r4^ operation	GB, IDH-wildtype; (recidive)	4	p53 positivity, no EGFR amplification	Chemoradiation therapy
GB2	M	(80–84)	primary	GB, IDH-wildtype;	4		naive
GS	M	(70–74)	primary	Gliosarcoma, IDH-wildtype;	4	p53 positivity, EGFR amplification,	naive
ODG	F	(45–49)	primary	Oligodendroglioma, IDH-mutant; 1p/19q co-deleted	2	none	naive
BrM L1, L2	F	(40–44)	primary	Carcinoma, metastatic site (breast cancer BrMs)	N/A	Estrogen receptor positive	Chemotherapy (catesitapin)

*GB* glioblastoma, *reGB* recurrent glioblastoma, *GS* gliosarcoma, *ODG* oligodendroglioma; BrM, brain metastasis, *NOS* non-otherwise specified, *IDH* isocitrate dehydrogenase, *WHO* World Health Organization, *EGFR* epidermal growth factor receptor, *GFAP* glial fibrillary acidic protein, *N/A* not applicable, Evaluated 2018

Samples were natively processed, magnetically sorted for CD45^+^ cell fraction and analyzed using FCM. These samples composed our in-house FCM cohort. Altogether, 259,636 CD45^+^ singlet events (i.e., cells) were collected and measured. Samples were also analyzed with transcriptome sequencing (RNA-seq).

Sequential bivariate density plots based on CD66b^+^ and CD3^+^ markers of FCM data (Fig. 1A) outlined four main subpopulations from CD45^+^ cells, including cells coexpressing CD66b^+^ and CD3^+^ (CD45^+^/CD66b^+^/CD3^+^ nonclassical cells), exposing inherent CNS immune complexity. Furthermore, the frequencies of T lymphocytes (CD45^+^/CD3^+^) strikingly differed between samples, ranging from 9% (oligodendroglioma) to 36% (gliosarcoma).

An unsupervised computational approach was used to cluster CD45^+^-enriched cells based on the FCM data from all patients combined. Briefly, surface marker fluorescent intensities (FIs), intrinsic cell features (size, FSC-A and granularity, SSC-A), and viability stain, all considered separate dimensions, were used as inputs for clustering with PhenoGraph [13] (see Methods). This generated phenotypic dissection of CD45^+^ cells into 25 clusters (Fig. 1B, Supplementary Fig. 1B). The resulting clusters were annotated based on marker, size, and granularity distributions, leading to the proposed phenotypes (see Methods). Thus, immune cells, i.e., cell phenotypes, were defined based on the combination of these parameters and their relative expression, which were henceforth referred to as phenoclusters. Subsequently, phenoclusters that indicated immune subsets of biologically coherent subpopulations were grouped under similar cell type categories (Fig. 1B, Supplementary Table 1).

Clustering analysis identified a diverse landscape of immune cell phenotypes. Visualization with t-distributed stochastic neighbor embedding (t-SNE) shows three main discernible regions (Fig. 1B): neutrophils, lymphoid phenoclusters (DNTs: CD45^+^/CD14^+^/CD3^+^ or CD45^+^/CD14^−^/CD3^+^, T helper cells: CD45^+^/CD3^+^/CD4^+^, and cytotoxic T cells: CD45^+^/CD3^+^/CD8^+^) and a group of MG and MDM phenoclusters, thus reflecting their broad lineage similarity (Fig. 1B, entire marker sets in Supplementary Table 1).

### Heterogeneous leukocyte composition across brain cancers

**Fig. 2:**
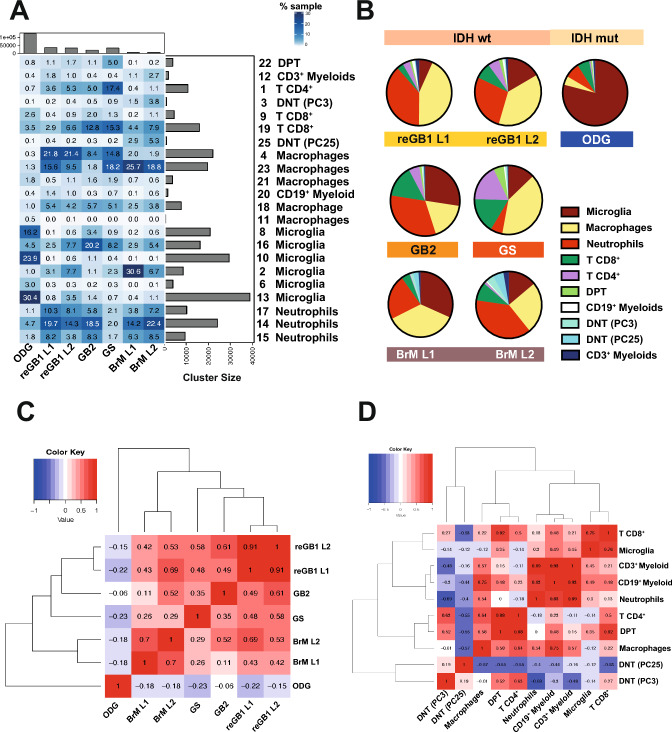
Unsupervised clustering identifies distinct leukocyte accumulations across brain cancers. **A** Cell phenotype frequencies are generally similar among similar tumor types. CD3^+^ myeloid, CD19^+^ myeloid, and double-negative T cells are present across samples. Proportions of cells in the discovered phenoclusters. Columns indicate tumor samples. Bar plots show the sample size (total number of CD45^+^ events) (top) and cluster event number (right). Numbers before cell phenotypes refer to the PhenoGraph clusters. ODG, oligodendroglioma; reGBM, recurrent glioblastoma; GBM, primary glioblastoma; GS, gliosarcoma; BrM, brain metastasis; L1, tumor locus 1; L2, tumor locus 2. **B** Microglia are predominant in the oligodendroglioma sample, whereas GBs and metastatic samples show higher macrophage and neutrophil frequencies. The highest DNT proportions (PC3, PC23) were detected in metastatic tumors. Pie charts representing the samplewise proportions of cell types in the FCM data; PC, phenocluster. **C** Oligodendroglioma (ODG) has the most distinct immune cell proportions, whereas different loci from the same tumors show the highest similarity. Heatmap visualizing the pairwise correlations between sample-based phenocluster distributions. **D** The frequencies of noncanonical myeloids (CD3^+^ myeloids and CD19^+^ myeloids) closely correlate across samples. Heatmap showing cell typewise correlations to explore associations between population subsets; DPT, double-positive T cells (CD3^+^CD4^+^CD8^+^); DNT (PC25), double-negative T cells (CD3^+^CD4^−^CD8^−^CD14^−^); DNT (PC3), (CD3^+^CD4^−^CD8^−^CD14^+^); PC, phenocluster

We compared TiME landscapes between different samples (Fig. 2A), and despite event number differences (Fig. 1C), a tumor type-specific gradient on infiltrating (neutrophils and MDMs) or resident myeloid MG cells was observed. For instance, the frequency of MG in IDH1^wt^ gliomas (12–27%) together with BrMs (14–34%) contrasted with the IDH1^mut^ oligodendroglioma (ODG) having the largest (− 79%) MG component (Fig. 2C). Similarly, infiltrating neutrophils were most abundant in BrMs (24–38%) and IDH1^wt^ gliomas (25–37%), except for IDH1^wt^ gliosarcoma (GS) (− 6%), with a similar frequency to IDH^mut^ ODG (− 8%). Samples obtained from two separate loci of the same tumor were most similar to each other, although BrM L2 also showed similarity with reGB1 L1 (Spearman correlation: 0.7, p-value = 0.0002). Again, the ODG sample showed the most divergent TiME. Based on immune cell proportions, samples were clustered according to tumor diagnosis (Fig. 2C), supporting the previously reported malignancy type-specific TiME [7, 8, 27].

Infiltrating lymphocyte fractions were highest in GS (CD8 T cells: 15%, phenocluster 19 and CD4 T cells: 17%, phenocluster 1), even in comparison with BrMs (CD8 T cells: 4–9%, CD4 T cells: − 0.4–1%), which are known to bear sizeable CD8^+^ T fractions [7, 8, 27]. The GS sample also carried the highest proportion of double-positive T cells (CD66b^+^/CD45^+^/CD14^+^/CD3^+^/CD4^+^/CD8^+^, phenocluster 22) (Fig. 2B). Compatible with this active TiME landscape, GS showed the highest number of different gene fusion transcripts (indicative of chromosomal rearrangements predominantly in chromosomes 12 and 14, Supplementary Table 2) compared to the other in-house FCM tumor samples.

Despite their overall myeloid profile (CD45^+^/CD19^+^/CD14^+^, phenocluster 20 and CD66b^+^/CD45^+^/CD14^+^/CD3^+^, phenocluster 12), phenocluster 20 exhibited CD19^+^ positivity (with cluster stability-Jaccard coefficient over 0.75; Supplementary Fig. 2B, C), while phenocluster 12 presented CD3^+^ positivity (Supplementary Fig. 2C). Consequently, phenoclusters 20 and 12 were annotated as noncanonical myeloid subsets: CD19^+^ and CD3^+^ myeloids, respectively (Fig. 2A). Furthermore, we detected T lymphocyte phenoclusters [PC] that were negative for CD4 and CD8 markers (phenoclusters 3 and 25) and annotated them as DNTs (Fig. 2A). Due to cluster instability, phenocluster 3 was not considered further (DNT [PC3], Supplementary Fig. 2B).

Even though the CD45^+^ cell counts differed between samples, CD3^+^ myeloids, CD19^+^ myeloids, and DNTs were present across all samples (Fig. 2B) and represented a small but robust subset of immune cell types (Fig. 2B).Additionally, CD4 and CD8 markers had a bimodal distribution in CD3^+^ myeloid cells (phenocluster 12, Supplementary Fig. 2C), suggesting the possibility of further subpopulations within this cell subset.

To further stratify the TiME based on phenotype categories, correlations between cell type (i.e., groups of phenoclusters with biologically coherent subpopulations) proportions were explored. Noncanonical myeloids were highly correlated with each other (Pearson correlation: 0.93, p-value = 0.0026), followed closely by neutrophils (CD19^+^ myeloid: 0.83, p-value = 0.021; CD3^+^ myeloid: 0.89, p-value = 0.0066) and MDMs (CD19^+^ myeloid: 0.75, p-value = 0.051; CD3^+^ myeloid: 0.57, p-value = 0.17), endorsing their myeloid profile (Fig. 2D). Interestingly, DNT (PC25) displayed the most distinct frequency distribution compared to other immune subsets, while double-positive T cells (DPT) correlated with CD4 T cells (correlation: 0.88, p-value = 0.0094) and CD8 T cells (correlation: 0.82, p-value = 0.0227), suggesting their concomitant accumulation with other T lymphoid cells (Fig. 2D).

### Analysis of CD19^+^ myeloid and DNT cells in external mass cytometry data

**Fig. 3:**
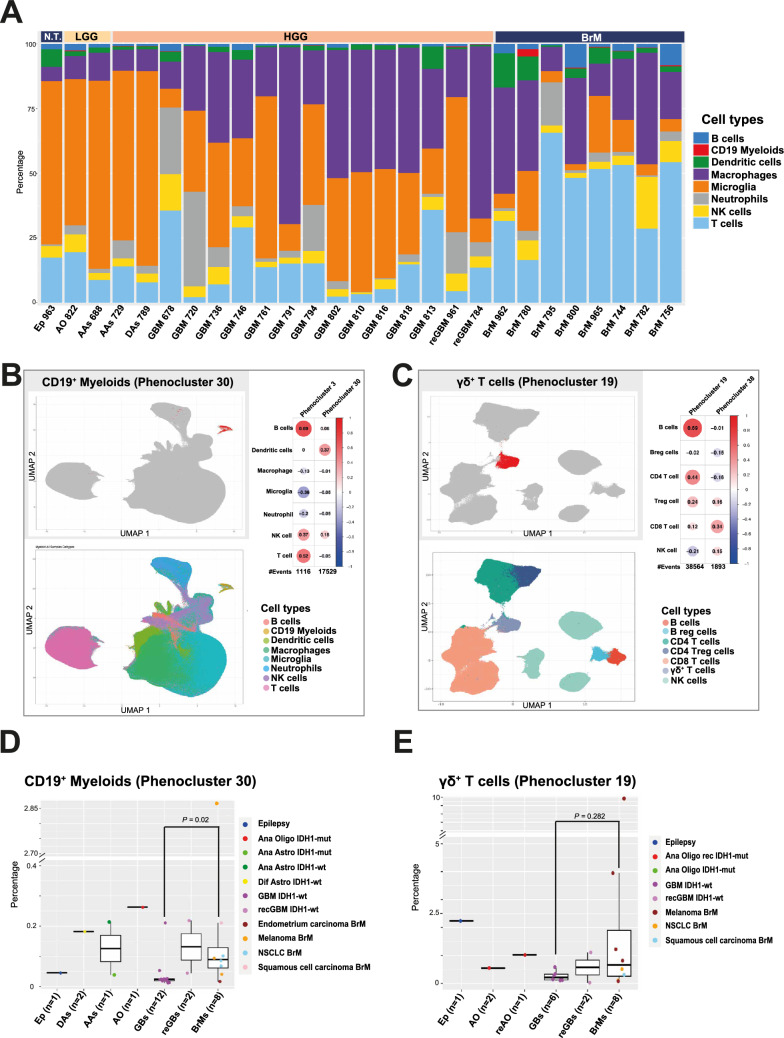
CD19^+^ myeloid and DNT cells validated in external mass cytometry data. **A** CD19^+^ myeloids detected in all samples. Stacked bar plot visualization of CD45^+^ events from 26 individuals showing the cell phenotypes based on cell type identification after unsupervised clustering, shown per patient sample. The results are based on CyTOF myeloid antibody panel data; N.T., nontumorous; LGG, lower-grade glioma; HGG, higher-grade glioma; Ep, epilepsy; AO, anaplastic oligodendroglioma; AA, anaplastic astrocytoma; DA, diffuse astrocytoma; GB, glioblastoma; reGB, recurrent glioblastoma; BrM, brain metastasis. **B** CD19^+^ myeloid cells form a separate cluster in UMAP visualization (top; UMAP, uniform manifold approximation and projection). UMAP visualization of cell types in the myeloid-focused panel data. Altogether, 9,778,719 CD45^+^ cells were included in the analysis (bottom; for CD3^+^ myeloids, see Supplementary Fig. 3). Cells in the main CD19^+^ myeloid phenocluster tend to coaccumulate with dendritic cells. Correlation analysis (right) between the frequencies of CD19^+^ myeloid clusters and other cell types across samples in the myeloid-focused panel data. **C** The CyTOF lymphoid antibody panel suggests *γδ* T cells as a suitable phenotype for DNTs. DNTs (CD3^+^CD4^−^CD8^−^) (CyTOF phenocluster 19, in red), also expressing *γδ*^+^ T-cell markers, are clearly separated from other T cells. UMAP visualization of cell types in the lymphoid-focused panel data (bottom). Altogether, 1,700,546 cells were included in the analysis. Cells in the main *γδ* T-cell cluster coaccumulate with B and CD4 T cells. Correlation analysis (right) between the frequencies of *γδ* T-cell clusters and other cell types across samples in the lymphoid-focused panel data. **D** BrMs accrue higher CD19^+^ myeloid frequencies than primary glioblastomas, with outlier frequency detected in a melanoma BrM (ZH780). Box plots showing proportions of CD19^+^ myeloid cells (CyTOF phenocluster 30) within the CD45^+^ cells in the myeloid-focused CyTOF panel dataset. NSCLC, non-small cell lung cancer; p < 0.05, two-sided Wilcoxon test. **E** Lower *γδ*^+^ T-cell frequencies detected generally in GBs, with an outlier frequency in a melanoma (ZH800). Box plots showing the frequencies of *γδ*^+^ T cells (CyTOF phenocluster 19) in the lymphoid-focused panel dataset

Intrigued by our novel myeloid phenotypes, we analyzed an external dataset to interrogate and validate our findings. For this, we utilized the single-cell mass cytometry by time of flight (CyTOF) dataset from [7], enquiring the immune cell fractions of brain tumor samples by using antibody panels designed for myeloid (26 patients) or lymphoid (18 patients) cells (Supplementary Table 2, 40 different patients in total). The myeloid-focused panel included the CD45 marker, permitting us to carry out a similar CD45^+^ population-focused immune cell stratification with PhenoGraph as performed with in-house FCM data (Fig. 3A). Similar overall immune cell-type accumulation trends were observed across samples: resident MG enrichment in IDH1^mut^ gliomas (− 56–73%) and nontumorous controls (63%)[7, 8], while MDM proportions in IDH1^wt^ gliomas ranged from 8 to 68% [6–8]. Concordant with our FCM results, immune subset frequency patterns were also observed in the external FCM dataset (4 patients) [7] (Supplementary Fig. 3 upper panel). In the CyTOF myeloid-focused dataset (Fig. 3A), the phagocyte compartment was further dissected, including dendritic cells (DCs) (Fig. 3B lower panel), including several DC phenoclusters with various phenotypes while sharing typical DC-associated markers (e.g., CD11c, CD1c and CCR2), thus revealing the rich myeloid subset composition in CNS TiME. Furthermore, the extended antibody panel allowed phenotype definitions based on surrogate markers in the absence of robust subset markers [7, 9, 28].

The presence of a CD19^+^ myeloid cell population was confirmed with CyTOF data. These cells clustered separately in the UMAP visualization (Fig. 3B upper panel) and formed two phenoclusters (Clusters 3 and 30). They were significantly more abundant in BrMs than in primary GB (Fig. 3D). CD19^+^ myeloid frequencies (phenocluster 30) correlated (Pearson correlation 0.37) with DCs (Fig. 3B, right).

A unique phenocluster was not detected for CD3^+^ myeloids in the CyTOF data. However, within CD19^+^/CD14^+^/CD66b^+^ cells in the UMAP visualization, there was a subset of cells positive for the CD3^+^/CD14^+^/CD66b^+^ marker, potentially representing CD3^+^ myeloid cells (Supplementary Fig. 3 lower panel, B).

The lymphoid-focused CyTOF panel revealed the presence of *γδ* T cells (CD3^+^/*γδ*TCR^+^, Supplementary Table 2) (Fig. 3C). This cell subset was positive for the CD3 marker but negative for CD4 and CD8 (Fig. 3C upper panel), and it was at higher frequencies in BrMs (10% in melanoma BrM, Fig. 3E). These cells were consistent with our PC25 DNTs (Fig. 2A). *γδ* T cells were clearly concomitant with B cells (Pearson correlation 0.69), followed by CD4 T cells (Pearson correlation 0.44) (Fig. 3C, right).

### Transcriptome-based characterization of the tumor immune microenvironment supports phenotypes obtained from flow cytometry data

**Fig. 4:**
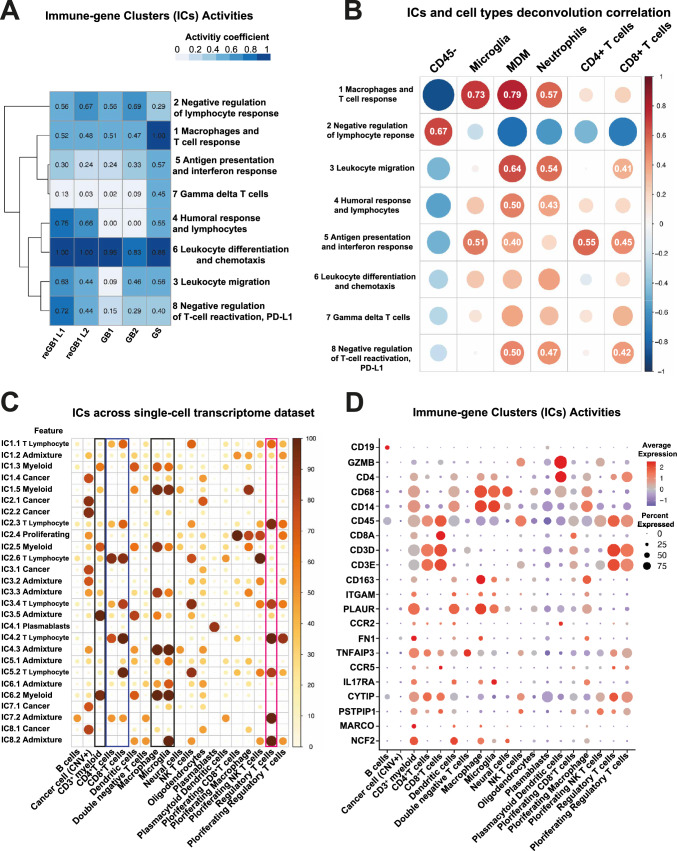
Transcriptional analysis of TiME supports phenocluster distributions in tumors characterized with FCM. **A** The immune-gene cluster activity patterns describe sample TiMEs. Expression data from a subset of cell suspension samples used for the flow cytometry analysis were analyzed with RNA-seq. The GB1 sample is a matched primary tumor sample for reGB1 L1 and L2. GB, glioblastoma; reGB, recurrent glioblastoma; GS, gliosarcoma. **B** MDM and neutrophil accumulation are linked to the negative regulation of T-cell activation (IC8) and leukocyte migration (IC3). Circle plots of Spearman correlation between frequencies of deconvoluted reference cell types (columns) and immune-gene cluster activities (rows) across TCGA and our RNA-seq data. Positive correlation coefficient values exceeding 0.4 are marked into the figure. **C** Expression patterns of immune cluster (IC) genes show distribution of genes into cell type categories in GB single-cell transcriptome data. The fraction of genes overexpressed within each IC subcluster is shown separately for each cell type. Prominent cell type expression pattern is indicated after subcluster ID (e.g., IC1.1 T lymphocyte). **D** In GBM single-cell transcriptome data, CD3^+^ myeloids share characteristics with dendritic cells while expressing CD3 markers. Dot plot showing marker gene expression patterns for neoplastic and immune cell types. Clustering revealed a CD3^+^ myeloid subset compatible with the flow cytometry phenotype in our analyses. Dot sizes indicate the percentage of cells expressing the gene, and colors indicate the average expression levels. NK-T cells, natural killer T cells; CNV, copy number variation

We used gene expression analysis to study immune system-related responses, as described previously [15], within the in-house FCM cohort (Fig. 1A). The analysis utilizes immune response-related gene clusters composed of genes that correlate across the TCGA GB cohort, thus suggesting a direct or indirect relationship. Based on the activity (i.e., median expression) of immune-gene clusters (ICA, see Methods) (Fig. 4A), the substantial infusion of leukocytes (lymphoid and myeloid) in GS tumor (Fig. 2B) was supported by the ICA profile. GS remarkably differed from the other tumors with respect to the activities of immune-gene Cluster 1 (IC1, macrophage and T-cell response, highest ICA: 1.00), IC5 (antigen presentation and interferon response, highest ICA: 0.57) and IC7 (gamma delta T cells, highest ICA: 0.45), corresponding with FCM results that show immune activity and strong accumulation of macrophages and T cells in this tumor (Fig. 2B). Furthermore, the GS tumor also showed DNT cell frequencies (Fig. 2A). In contrast, the primary tumor of GB1 showed lower ICAs in immune-gene Clusters 3 (leukocyte migration), 4 (humoral response and lymphocytes), and 8 (negative regulation of T-cell reactivation, PD-L1) when compared to tumor recurrences (reGB1 L1 and L2), suggesting adaptive immune responses in recurrent TiME samples.

Next, we utilized deconvolution and previously generated cell type references [8] to estimate the relative cell type proportions (see Methods) and analyzed how they correlate with the ICAs after combining our samples with the TCGA GB cohort (Fig. 4B). IC2 (negative regulation of lymphocyte response) negatively correlated with all the immune cell type proportions, whereas IC1 (macrophages and T cell response) showed positive correlation to microglia, MDM, and neutrophils. Furthermore, IC8 (negative regulation of T-cell reactivation, PD-L1) and IC3 (leukocyte migration) were associated with MDM, neutrophil and T CD8 cell proportions (Fig. 4B).

For single-cell transcriptome data analysis, we compiled SCS data from four publications (see Methods), resulting in a unified dataset from 28 patients [9, 18–20]. Clustering analysis revealed classical immune cell phenotypes and a cell subset compatible with CD3^+^ myeloids.

To explore the immune-gene cluster (IC) gene expression at single-cell level, the fraction of overexpressed genes in each IC subcluster (defined computationally based on overexpression pattern in SCS data) was calculated separately for each cell type (see Methods, Fig. 4C). This revealed the distribution of genes across cell types in all the ICs, which could be further categorized into expression patterns that are predominant in cancer cells, myeloid cells, T lymphocytes, or plasmablasts or show an admixture distribution. The results provided support to the original naming of the clusters. For example, IC1 (macrophages and T cell response) included genes overexpressed in myeloid cells, cancer, or T lymphocytes, although the activity of this cluster is not associated with T cell accumulation (Fig. 4B). The majority of genes (830 out 1067, 78%) in IC2 (negative regulation of lymphocyte response) were overexpressed in cancer cells, but genes in subcluster IC2.3 were overexpressed in T lymphocytes. IC5 (antigen presentation and interferon response), whose activity positively correlated with T cell and microglia proportions (Fig. 4B), included a subset of genes (IC5.2) with a clear overexpression in T lymphocytes and a subcluster IC5.1 with a highest proportion of overexpressed genes in microglia.

Over 50% of the genes were overexpressed in CD3^+^ myeloids in subclusters IC1.3, IC2.5, IC3.5, and IC6.2 (Fig. 4C; Supplementary Fig. 5B). All these subclusters showed a myeloid cell overexpression pattern. Also, when looking at the individual markers, CD3^+^ myeloid cluster (Fig. 4D) showed the expression of myeloid markers (CD14, CD68, MARCO, IL17RA, ITGAM) (Fig. 4D). However, CD3^+^ myeloids also expressed lymphoid markers (CD3D, CD3E) (Fig. 4D), distinguishing them from the other myeloid subsets. Thus, CD3^+^ myeloids substantiated their myeloid profile, sharing characteristics with DCs while expressing T lymphocyte markers.

We examined the possible role of CD3^+^ myeloid cells in TiME by ORA of differentially expressed genes comparing CD3^+^ myeloid cells with all other non-neoplastic cells combined. ORA downregulated pathways include evident suppression of cellular immune response (e.g., “leukocyte mediated immunity”, “positive regulation of leukocyte activation” and “complement activation”; Supplementary Fig. 4A), while upregulated pathways include (e.g., “taxis”, “granulocyte chemotaxis” and “humoral immune response”; Supplementary Fig. 4A).

To further investigate the role of CD3^+^ myeloids within the TiME compartment, ORA comparisons between different immune cell types (among SCS clusters) were made. The ORA between CD3^+^ myeloids and DCs showed downregulation of cell activation processes and antigen presentation via major histocompatibility complex (MHC) class II in CD3^+^ myeloids (e.g., “antigen processing and presentation”, “MHC class II protein complex assembly” and “phagocytosis”, Supplementary Fig. 4B). These findings concurred with the ORA analysis between CD3^+^ myeloids and T lymphocytes (T CD4 or T CD8, Supplementary Fig. 4C-D), showing downregulation of T-cell activation (e.g., “regulation of lymphocyte activation” or “regulation of T-cell activation”, respectively). Analogously, CD3^+^ myeloid ORA upregulated genes included (e.g., “cell killing”, CD3^+^ myeloids vs. DCs) or myeloid activation pathways (e.g., “myeloid leukocyte activation”, CD3^+^ myeloids vs. T CD4 or vs. T CD8, Supplementary Fig. 4 C-D). Taken together, these features suggest that CD3^+^ myeloids are a separate entity with a role in immune regulation.

As a further validation, we sought to find the presence of noncanonical myeloids in intact CNS tumor tissues with multiplex-IHC staining. For this purpose, we used a validation cohort comprised of 14 samples from nine glioma including three diffuse IDH^mut^ astrocytomas, on two IDH^mut^ oligodendroglioma, 1p/19q co-deleted, and four GBM IDH^wt^ tumors. For four patients, both primary and recurrent samples were available (one diffuse astrocytoma and three GBM IDH^wt^ patients; Supplementary Table 4). We immunostained these patient samples with antibodies typical for noncanonical immune cell subsets. In six tumors from five patients, we observed evidence of immune cells (CD45 positive) at very low frequencies (in line with FCM, CyTOF and SCS analyses) with co-localization of myeloid- and lymphoid-associated receptors on the same single cell, indicating a noncanonical myeloid phenotype (markers stained for were CD45, TMEM119, CD68, CD11c, CD3; Supplementary Fig. 6). These markers were present at different combinations endorsing the notion of subpopulations with a novel cell phenotype (Supplementary Table 4). Furthermore, for DNTs (namely CD45^+^/CD3^+^/CD4^−^/CD8^−^ T lymphocytes; DNTs were not analyzed in the GBM IDH^wt^ tumors) immunostaining also confirmed evidence of DNTs in all the analyzed IDH^mut^ diffuse astrocytoma and oligodendroglioma tumors (Supplementary Fig. 7).

## Discussion

This study aimed to determine whether nonclassical immune subsets could be part of CNS TiME. The majority of the cells represented canonical cell types, and their frequencies followed the typical patterns for the tumor types involved [7, 8]. However, our data-driven approach was able to uncover novel noncanonical immune subsets. In line with preceding reports, IDH^WT^ gliomas had higher lymphocytes than IDH^mut^ gliomas, and we corroborated the substantial neutrophil component in CNS tumors [7, 8, 10, 27]. A GS tumor (a GB subtype) showed clearly elevated frequencies of T lymphocytes even compared to BrMs. Such T lymphocyte infiltration could be partially explained by the higher extent of gene fusions found in the GS tumor than in the other in-house tumor samples, indicative of elevated neoantigen presence in GS TiME.

Furthermore, unsupervised clustering compartmentalized our in-house FCM data T lymphocytes to include the CD14^−^/CD8^−^/CD4^−^/CD3^+^ T subset, DNT (PC25). Complementing these findings, the extended lymphoid-focused CyTOF dataset proposed a suitable phenotype for DNT (PC25), *γδ* T cells[7]. Their involvement in TiME regulation has been reported, as the neutrophil recruitment has been driven by *γδ* T cell-derived interleukin-17, and as *γδ* T cell depletion in mice has led to a marked reduction of secondary lymph node or pulmonary metastases from mice bearing mammary tumors [11]. On the other hand, the hypoxic brain TME has been reported to suppress tumor infiltrating *γδ* T cells and to induce their apoptosis also in avatar models [29].

The central findings obtained from unsupervised clustering analysis were two subsets of noncanonical myeloid CD3^+^ or CD19^+^ cells, which were present independent of tumor type. Thus, the presence of noncanonical myeloids suggested that TCR complex-related marker specificity could not be restricted to the T-lymphoid lineage, at least in CNS TiME. Surprisingly, a wealth of prior reports accounted for several myeloid subsets expressing traditionally lymphoid-restricted receptors: neutrophils from peripheral blood (subpopulation up to 5% of CD16^+^/TCR^+^, CD8^−^/CD4^−^/CD3^−^) expressed the T-cell receptor (TCR) [30], and CD14^+^ macrophages (subset of 5%) were TCRα^+^/TCRβ^+^ and expressed the major histocompatibility complex MHC class II [31]. These observations were also supported by TiME studies in which macrophages were TCRα^+^/TCRβ^+^ in esophageal cancer, colon cancer and colorectal carcinoma metastasis [32]. Interestingly, these noncanonical TCR^+^ myeloids did not express CD3 markers. However, in another study, peripheral blood CD14^+^ monocytes from healthy donors were differentiated into MDMs in vitro and gave rise to a CD3^+^ myeloid-compatible phenotype (− 15%), referred to as CD3^+^ MDMs [33, 34]. Moreover, in our analysis of the external SCS dataset, the macrophage receptor with collagenous structure (MARCO) was more highly expressed within the CD3 + myeloid cell cluster compared to other leukocyte cell types. Interestingly, MARCO^hi^ macrophages have been identified solely in mesenchymal GB tumors [35]. Similarly, the noncanonical myeloids detected in our multiplex-IHC validation cohort suggested an evident myeloid profile, including CD45, CD11c and CD68 markers which were detected together with CD3.

Regarding CD19^+^ myeloid cells, studies referring to classically lymphoid-considered functions reported that purified TAMs from melanoma patients expressed rearranged immunoglobulin chains, e.g., IgG and IgM [36]. In our view, this could provide grounds to not exclude ectopic immunoglobulin production, instead of aberrant expression [36]. Analogously, CD19^+^ plasmacytoid DCs precursors were found in a cohort of 301 pediatric patients with B-lymphoblastic leukemia, and although possibly differentiated from B-lymphoid cells, CD19^+^ plasmacytoid DCs were consistently negative for CD20 (B-cell marker) [37]. This observation could be in line with phenocluster 3 (CD19^+^ myeloid) in the external CyTOF dataset, showing canonical DC markers (e.g., CCR2, CD1c, CD11c and CD123) in addition to the CD19 marker, thus fitting the plasmacytoid DCs phenotype. The other CD19^+^ myeloid (phenocluster 30) did not have an evident DC-like marker pattern.

Alternatively, evidence of skull bone marrow-originated immature neutrophil precursors that differentiate into neutrophil-dendritic cell “hybrids” has been recently reported in human GB parenchyma. These hybrid TANs expressed canonical DC-related genes [38]. Similarly, CD3^+^ myeloids exhibited the CD66b marker both in our FCM cohort and in the CyTOF dataset, indicative of a neutrophil-like phenotype.

In this study, we investigated immune cell compartments across human tumors with data-driven approaches. Our FCM cohort comprised five patients with different tumor types. Provided the limited number of samples and number of antibodies included in FCM, we similarly assessed and verified our noncanonical subsets in external datasets, comprising a total of 68 patients (CyTOF cohort, myeloid panel: 26 patients; CyTOF cohort, lymphoid panel: 14 patients; SCS cohort: 28 patients). Moreover, we further confirmed the presence of noncanonical CD3^+^ myeloids and DNTs on the additional 5 diffuse glioma patients with using multiplex-IHC.

Our three main datasets (FCM, CyTOF and SCS) differed in several respects. Despite the limited marker set in the FCM cohort, it covered more lymphoid markers than the CyTOF myeloid-focused marker panel. CyTOF marker panels were extensive but focused either on myeloid or on lymphoid (lacking CD45) markers.

Differences in used antibodies as well as FCM and CyTOF methods need to be accounted for. SCS transcriptome data with thousands of measurement points also has issues with gene coverage in individual cells. Part of the markers, such as CD66b, are not included in all the data types. Furthermore, sample handling typically depletes neutrophils from SCS data and is likely to affect their frequencies also in other datasets. FCM data were generated from native tissue, immediately after surgery without freezing, likely to better preserve neutrophils. Together these aspects influence cell type representation and cell clustering approaches, which is a data-driven approach. For example, a separate CD3 myeloid cluster was not detected in CyTOF data although cells with a similar marker combination were present. The differences in these approaches, however, provide complementarity and support to the findings detected across datasets.

Moreover, with the current data, the origin or formation mechanisms of the noncanonical cell phenotypes remain to be studied. Lymphoid/monocyte doublets are an unlikely explanation as methodological (gating strategy with doublet exclusion) and data analysis approaches were taken to minimize cell doublets in our analyses. Furthermore, multiplex-IHC stains provided further evidence against the technical biases, such as cell doublets or classification issues. We cannot fully exclude biological processes, such as trogocytosis (acquisition of membrane from another cell), in their formation. Furthermore, immune cells are known to communicate with other cells via micro/tunneling nanotubes in the CNS TiME, which allows the exchange of cellular content, even organelles, between cells [39, 40]. SCS analysis showed that TCR complex genes are expressed within the CD3^+^ myeloid cluster, supporting the idea of cell-intrinsic expression of the marker genes, as endorsed by our results in the multiplex-IHC validation cohort. Our study centers on noncanonical subsets identification across brain tumors. Addressing their role through functional experiments that could provide mechanistic insights regarding their cell-of-origin and possible role remains to be explored. These questions will need to be addressed in future studies, including larger cohorts to confer solid grounds before generalizing them as a robust subset within CNS tumor immunobiology.

In summary, we have identified noncanonical myeloids within CNS TiME. We validated neutrophil enrichment as part of the classical myeloid compartment and provided insights into CD3^+^ or CD19^+^ myeloid subsets across brain tumor types. These noncanonical subsets were validated in external datasets and CD3^+^ myeloids in an in-house, multiplex-IHC validation cohort.

Taken together, our findings highlight the potential of unbiased data-driven approaches in resolving CNS TiME complexity. Future studies aiming to characterize noncanonical subsets are needed to reveal their functional role in CNS TiME. Moreover, a thorough investigation of noncanonical myeloid cells will be necessary to further discern their role in CNS TiME. Despite noncanonical myeloids being present at small frequencies, they can still have a relevant role in the brain tumor microenvironment, as shown in other instances [41, 42].

## Supplementary Information

Below is the link to the electronic supplementary material.

Supplementary file1 (PDF 380 KB)

Supplementary file2 (PDF 2630 KB)

Supplementary file3 (PDF 513 KB)

Supplementary file4 (PDF 162 KB)

Supplementary file5 (PDF 109 KB)

Supplementary file6 (PDF 388 KB)

Supplementary file7 (PDF 1693 KB)

Supplementary file8 (DOCX 12595 KB)

Supplementary file9 (XLSX 15 KB)

Supplementary file10 (XLSX 21 KB)

Supplementary file11 (XLSX 92 KB)

Supplementary file12 (XLSX 12 KB)

Supplementary file13 (DOCX 17 KB)

## Data Availability

Scripts were run using R packages available in github, link: https://github.com/NykterLab/Immune_infiltrates Normalized gene expression counts are deposited in the Gene Expression Omnibus (GEO) archive under the accession number GSE251900. The raw data from datasets generated during the current study are not publicly available due lack of patient consent that separately gives permission to this, but the corresponding author can be approached upon reasonable request. Meanwhile, accessing those records is possible with a GEO ID GSE251900 and a reviewer token “wdgjsusapjuphit”.

## Abbreviations

BrMs: Brain metastases
BSA: Bovine serum albumin
CNS: Central nervous system
CyTOF: Mass cytometry by time of flight
DCs: Dendritic cells
DE: Differential expression
DNTs: Double-negative T cells
pDC: Plasmacytoid dendritic cell
DPT: Double-positive T cells
EDTA: Ethylenediaminetetraacetic acid
FCM: Flow cytometry
FI: Fluorescent intensities
GB: Glioblastomas
GS: Gliosarcoma
ICA: Immune-gene clusters
IC: Immune-gene cluster
IDH: Isocitrate dehydrogenase
IDH^mut^: IDH-mutant gliomas
IDH^WT^: IDH wild-type
IL: Interleukin
MARCO: Macrophage receptor with collagenous structure
MDM: Monocyte-derived macrophages
MHC: Histocompatibility complex
MG: Microglia
ORA: Over-representation analysis
PBS: Phosphate-buffered saline
RNA-seq: Bulk-RNA sequencing
SCS: Single-cell RNA-seq
TAN: Tumor-associated neutrophils
TCGA: The Cancer Genome Atlas
TCR: T-cell receptor
TiME: Tumor immune microenvironment
t-SNE: T-distributed stochastic neighbor embedding
UMAP: Uniform manifold approximation and projection
WHO: World Health Organization
