# Supplementary figures and images for: Unsupervised clustering reveals noncanonical myeloid cell subsets in the brain tumor microenvironment
Source: Cancer Immunol Immunother. 2025 Jan 3;74(2):63. doi: 10.1007/s00262-024-03920-1 (PMC11699035; doi:10.1007/s00262-024-03920-1)

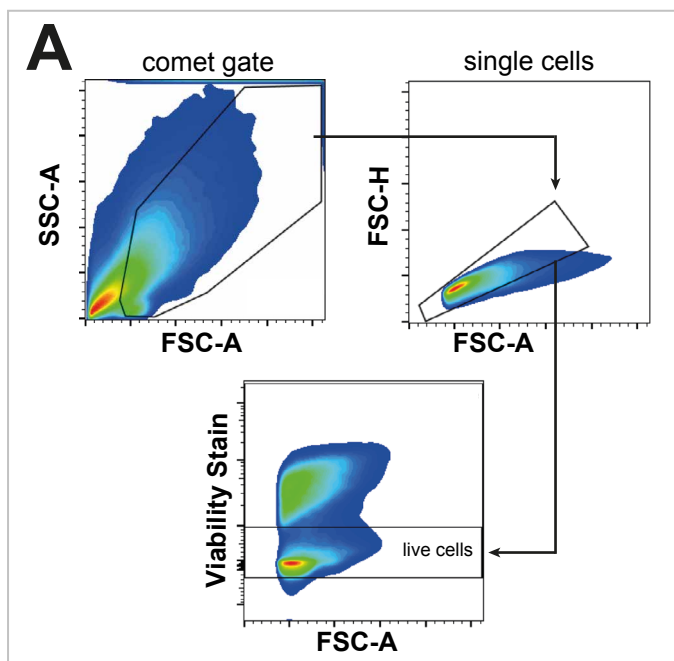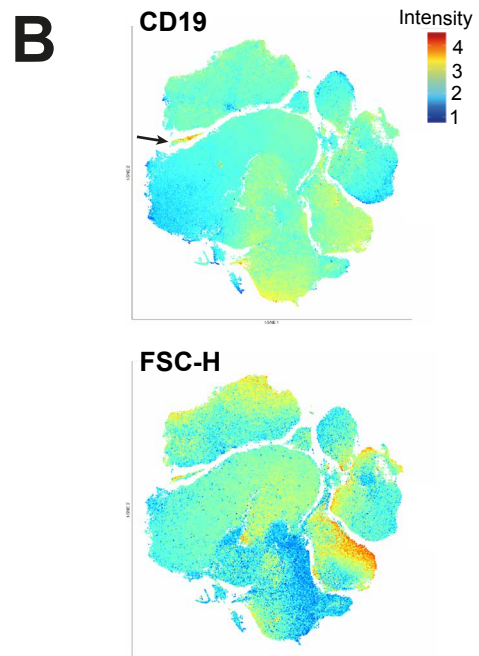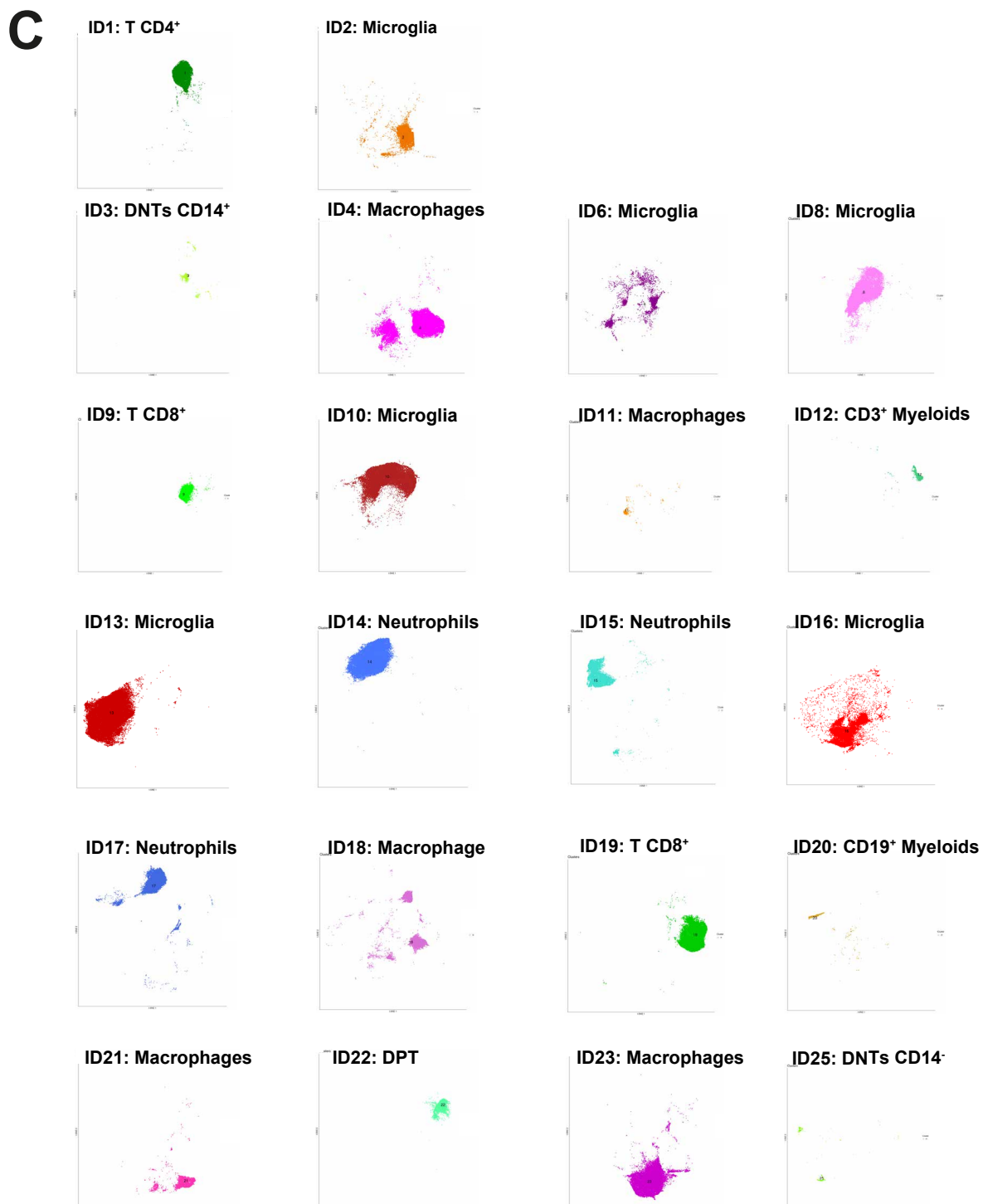

Supplement: Supplementary file 1 — Supplementary file1 (PDF 380 KB) [file 262_2024_3920_MOESM1_ESM.pdf]

A

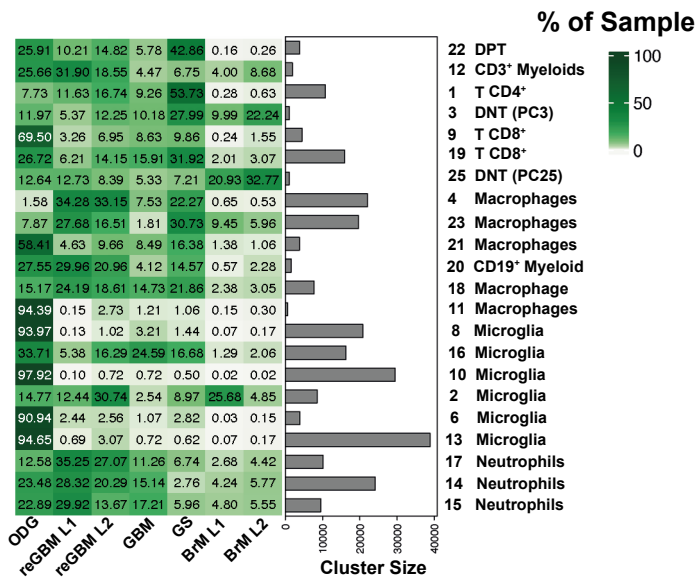

B

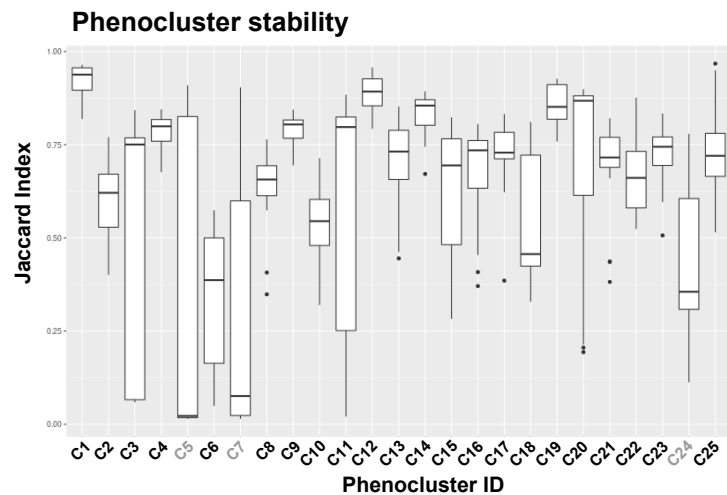

C

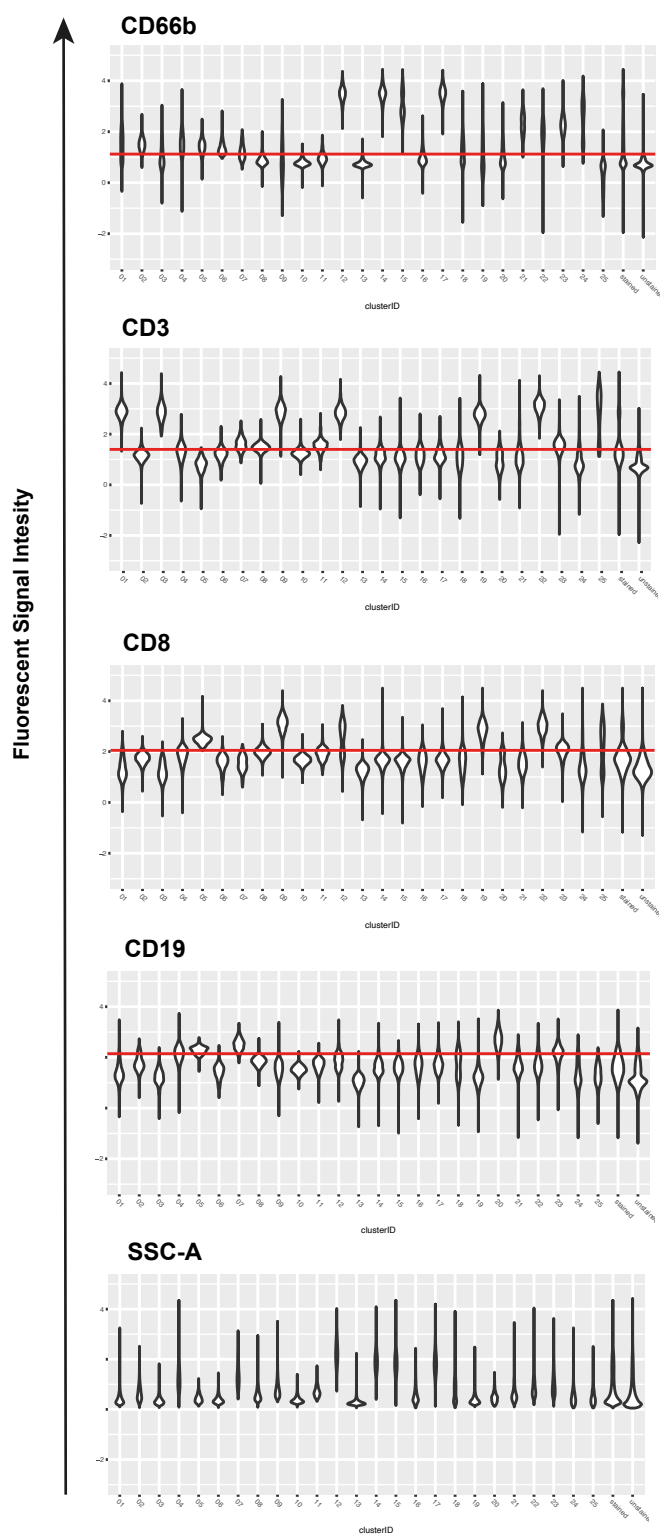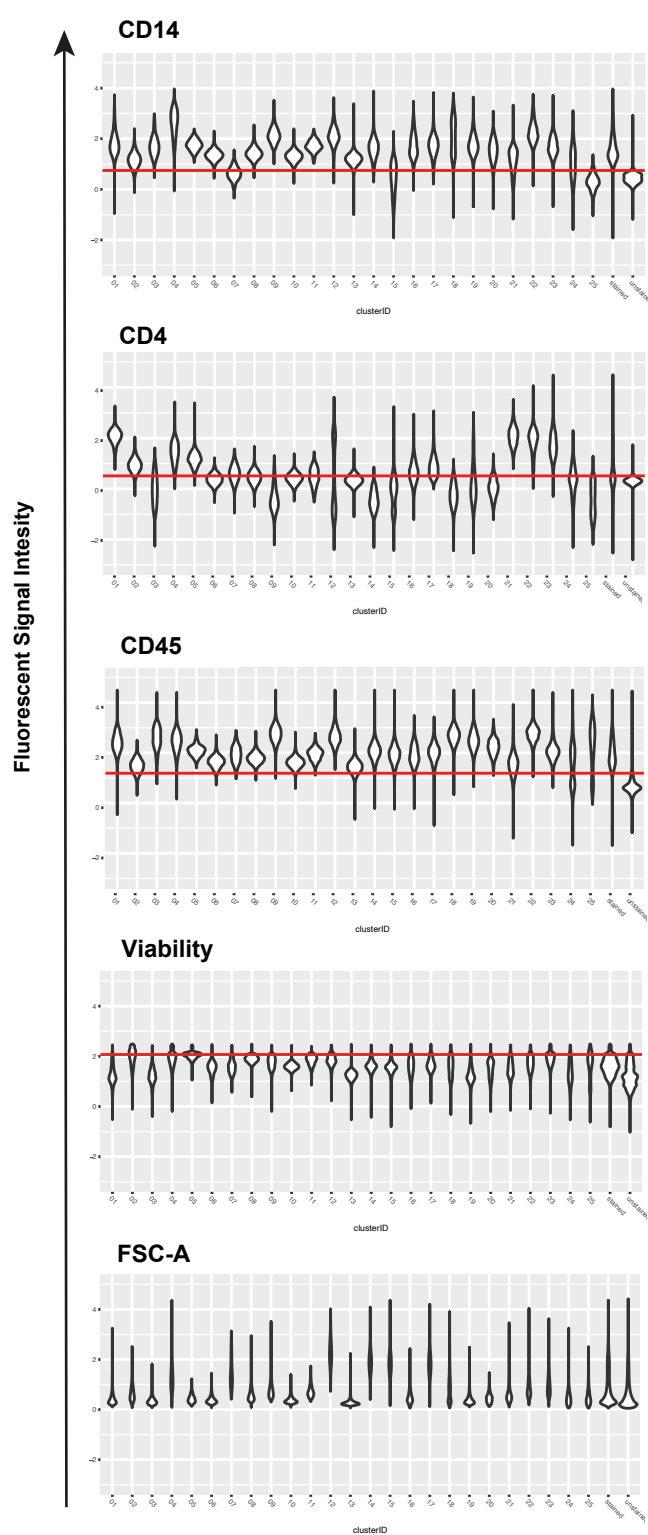

Supplement: Supplementary file 2 — Supplementary file2 (PDF 2630 KB) [file 262_2024_3920_MOESM2_ESM.pdf]

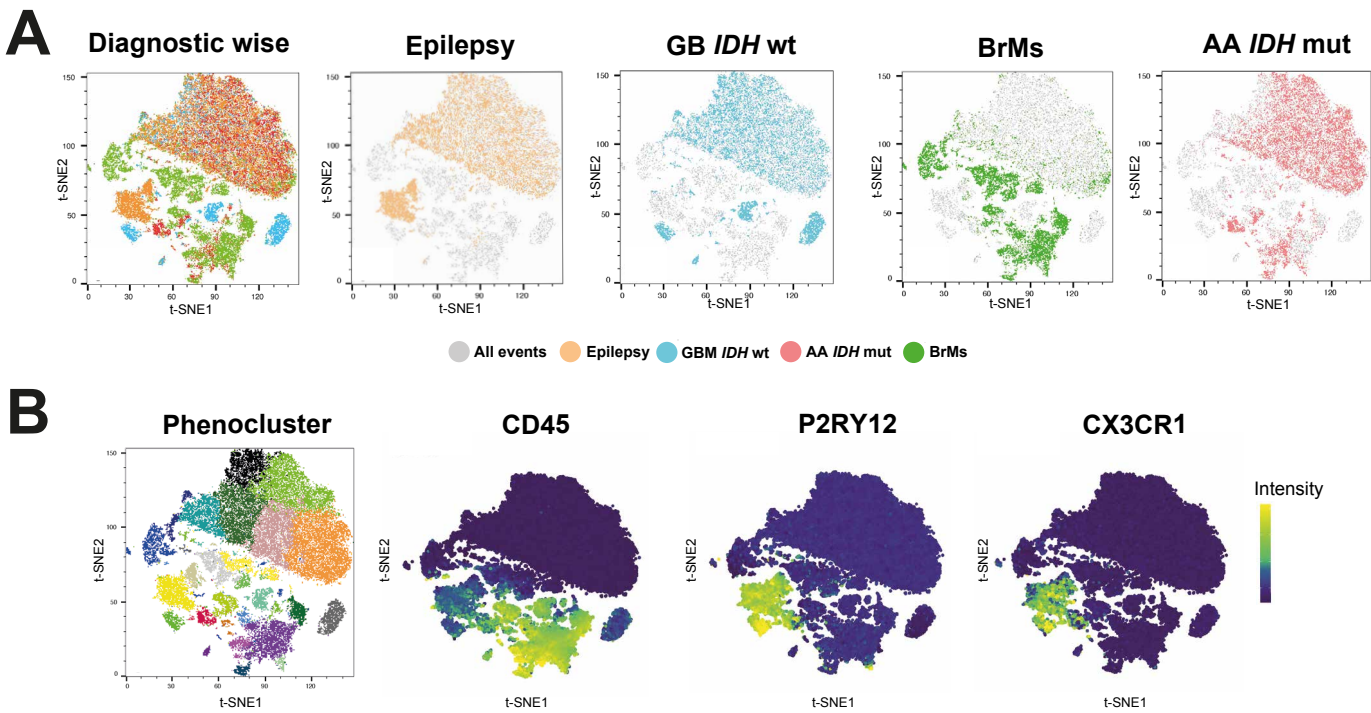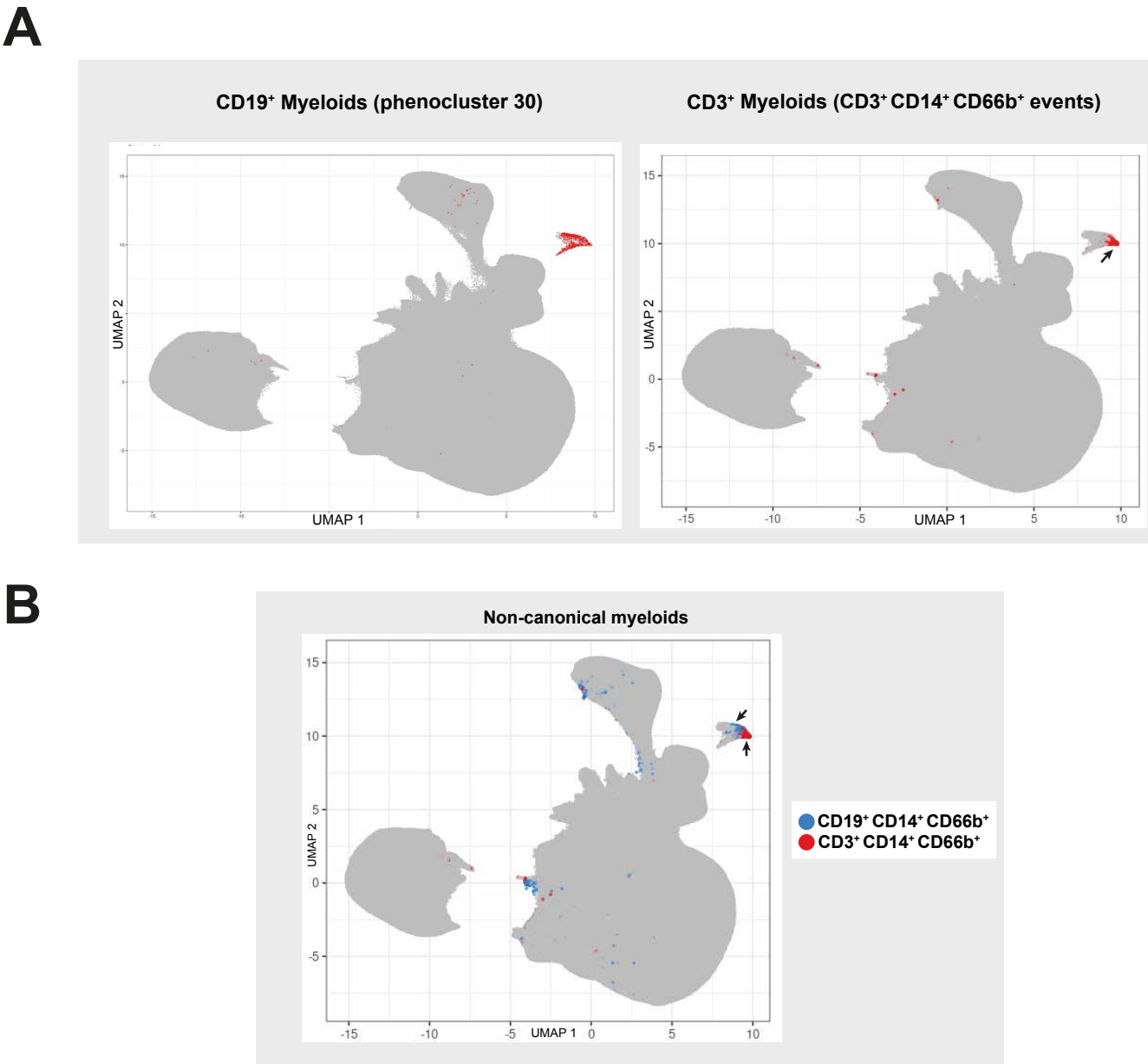

Supplement: Supplementary file 3 — Supplementary file3 (PDF 513 KB) [file 262_2024_3920_MOESM3_ESM.pdf]

A

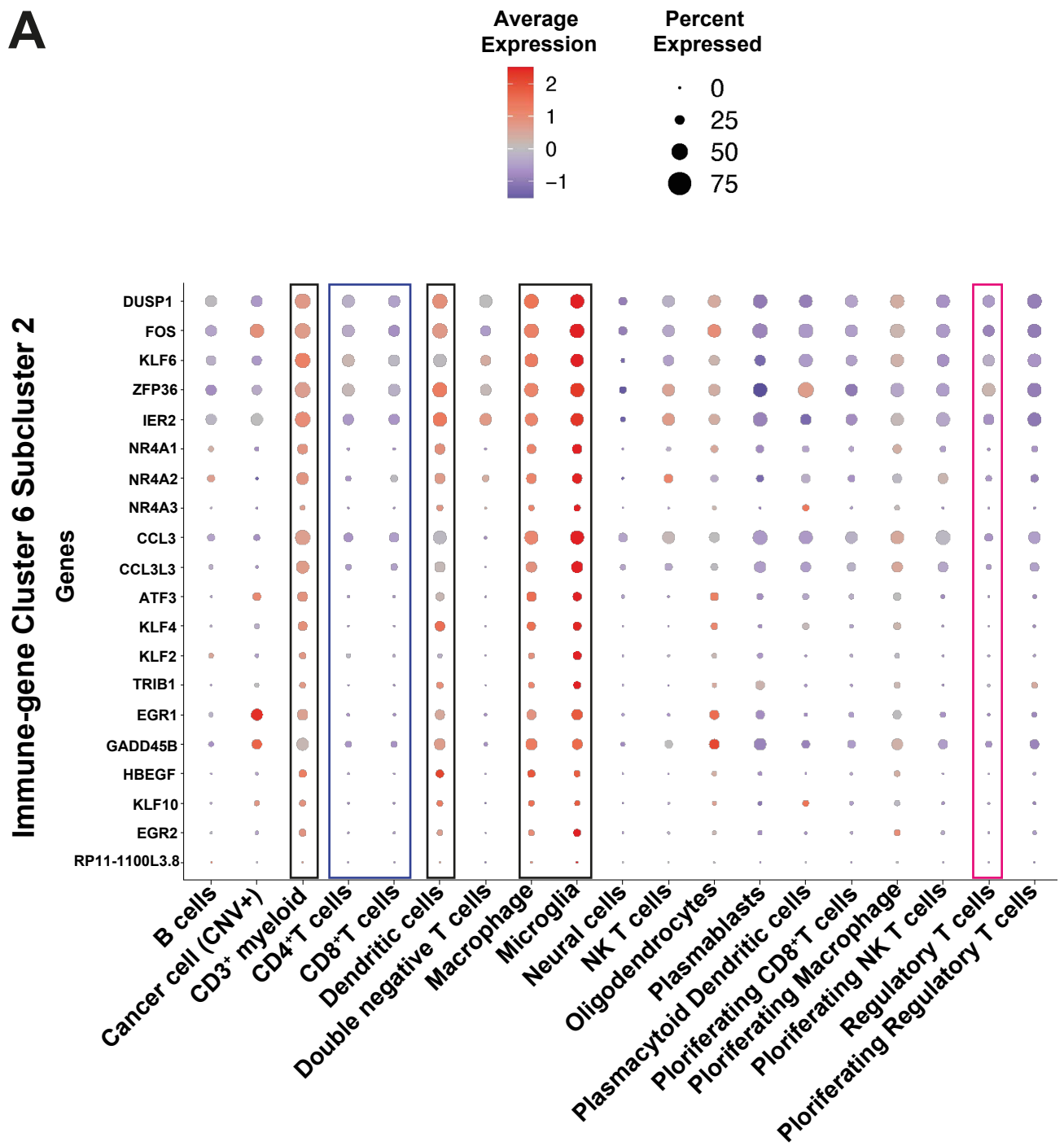

Supplement: Supplementary file 5 — Supplementary file5 (PDF 109 KB) [file 262_2024_3920_MOESM5_ESM.pdf]
