# Supplementary material for: Unsupervised clustering reveals noncanonical myeloid cell subsets in the brain tumor microenvironment
Source: Cancer Immunol Immunother. 2025 Jan 3;74(2):63. doi: 10.1007/s00262-024-03920-1 (PMC11699035; doi:10.1007/s00262-024-03920-1)
Supplement: Supplementary file 4 — Supplementary file4 (PDF 162 KB) [file 262_2024_3920_MOESM4_ESM.pdf]

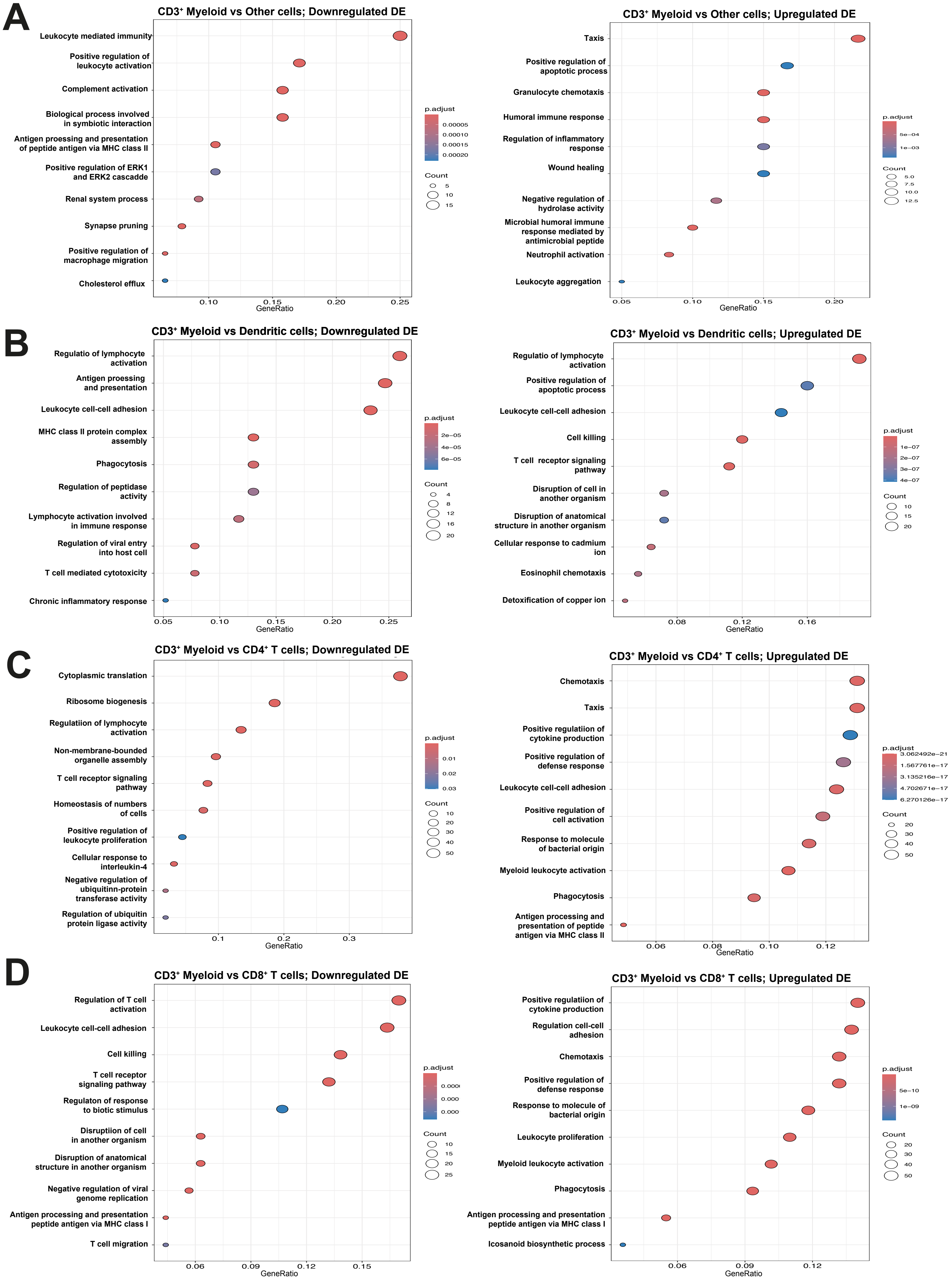
A
CD3+ Myeloid vs Other cells; Downregulated DE
Leukocyte mediated immunity
Positive regulation of leukocyte activation
Complement activation
Biological process involved in symbiotic interaction
Antigen proessing and presentation of peptide antigen via MHC class II
Positive regulation of ERK1 and ERK2 cascadde
Renal system process
Synapse pruning
Positive regulation of macrophage migration
Cholesterol efflux
0.10 0.15 0.20 0.25
GeneRatio
p.adjust
0.00005
0.00010
0.00015
0.00020
Count
5
10
15
CD3+ Myeloid vs Other cells; Upregulated DE
Taxis
Positive regulation of apoptotic process
Granulocyte chemotaxis
Humoral immune response
Regulation of inflammatory response
Wound healing
Negative regulation of hydrolase activity
Microbial humoral immune response mediated by antimicrobial peptide
Neutrophil activation
Leukocyte aggregation
0.05 0.10 0.15 0.20
GeneRatio
p.adjust
5e−04
1e−03
Count
5.0
7.5
10.0
12.5
B
CD3+ Myeloid vs Dendritic cells; Downregulated DE
Regulatio of lymphocyte activation
Antigen proessing and presentation
Leukocyte cell-cell adhesion
MHC class II protein complex assembly
Phagocytosis
Regulation of peptidase activity
Lymphocyte activation involved in immune response
Regulation of viral entry into host cell
T cell mediated cytotoxicity
Chronic inflammatory response
0.05 0.10 0.15 0.20 0.25
GeneRatio
p.adjust
2e−05
4e−05
6e−05
Count
4
8
12
16
20
CD3+ Myeloid vs Dendritic cells; Upregulated DE
Regulatio of lymphocyte activation
Positive regulation of apoptotic process
Leukocyte cell-cell adhesion
Cell killing
T cell receptor signaling pathway
Disruption of cell in another organism
Disruption of anatomical structure in another organism
Cellular response to cadmium ion
Eosinophil chemotaxis
Detoxification of copper ion
0.08 0.12 0.16
GeneRatio
p.adjust
1e−07
2e−07
3e−07
4e−07
Count
10
15
20
C
CD3+ Myeloid vs CD4+ T cells; Downregulated DE
Cytoplasmic translation
Ribosome biogenesis
Regulatiion of lymphocyte activation
Non-membrane-bounded organelle assembly
T cell receptor signaling pathway
Homeostasis of numbers of cells
Positive regulation of leukocyte proliferation
Cellular response to interleukin-4
Negative regulation of ubiquitinn-protein transferase activity
Regulation of ubiquitin protein ligase activity
0.1 0.2 0.3
GeneRatio
p.adjust
0.01
0.02
0.03
Count
10
20
30
40
50
CD3+ Myeloid vs CD4+ T cells; Upregulated DE
Chemotaxis
Taxis
Positive regulatiion of cytokine production
Positive regulation of defense response
Leukocyte cell-cell adhesion
Positive regulation of cell activation
Response to molecule of bacterial origin
Myeloid leukocyte activation
Phagocytosis
Antigen processing and presentation of peptide antigen via MHC class II
0.06 0.08 0.10 0.12
GeneRatio
p.adjust
3.062492e−21
1.567761e−17
3.135216e−17
4.702671e−17
6.270126e−17
Count
20
30
40
50
D
CD3+ Myeloid vs CD8+ T cells; Downregulated DE
Regulation of T cell activation
Leukocyte cell-cell adhesion
Cell killing
T cell receptor signaling pathway
Regulaton of response to biotic stimulus
Disruptiion of cell in another organism
Disruption of anatomical structure in another organism
Negative regulation of viral genome replication
Antigen processing and presentation peptide antigen via MHC class I
T cell migration
0.06 0.09 0.12 0.15
GeneRatio
p.adjust
0.000
0.000
0.000
Count
10
15
20
25
CD3+ Myeloid vs CD8+ T cells; Upregulated DE
Positive regulatiion of cytokine production
Regulation cell-cell adhesion
Chemotaxis
Positive regulation of defense response
Response to molecule of bacterial origin
Leukocyte proliferation
Myeloid leukocyte activation
Phagocytosis
Antigen processing and presentation peptide antigen via MHC class I
Icosanoid biosynthetic process
0.06 0.09 0.12
GeneRatio
p.adjust
5e−10
1e−09
Count
20
30
40
50
