# Supplementary material for: Unsupervised clustering reveals noncanonical myeloid cell subsets in the brain tumor microenvironment
Source: Cancer Immunol Immunother. 2025 Jan 3;74(2):63. doi: 10.1007/s00262-024-03920-1 (PMC11699035; doi:10.1007/s00262-024-03920-1)
Supplement: Supplementary file 6 — Supplementary file6 (PDF 388 KB) [file 262_2024_3920_MOESM6_ESM.pdf]

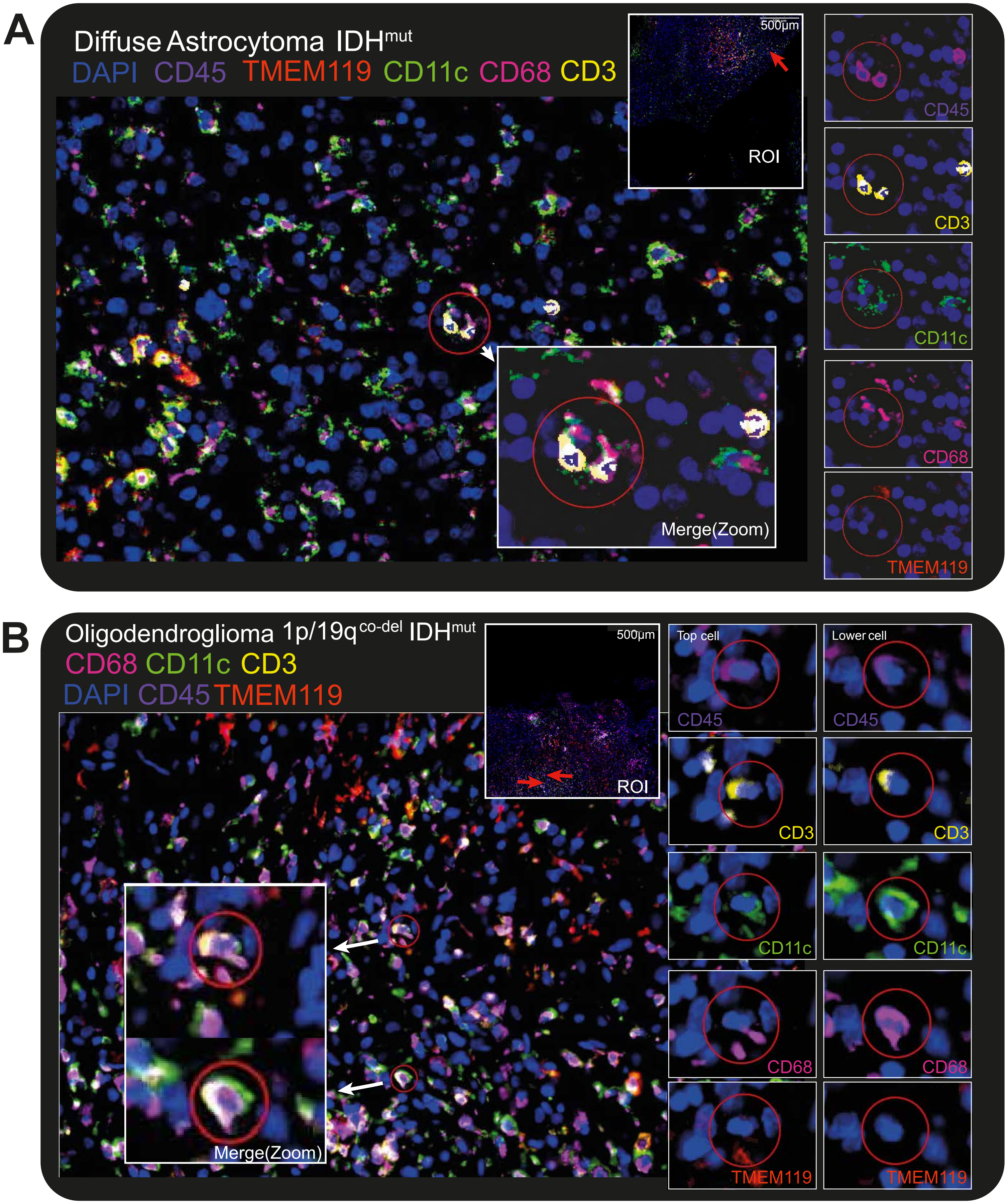
A
Diffuse Astrocytoma IDH^{mut}
DAPI CD45 TMEM119 CD11c CD68 CD3
500µm
ROI
Merge(Zoom)
CD45
CD3
CD11c
CD68
TMEM119
B
Oligodendroglioma 1p/19q^{co-del} IDH^{mut}
CD68 CD11c CD3
DAPI CD45 TMEM119
500µm
ROI
Top cell
Lower cell
CD45
CD3
CD11c
CD68
TMEM119
Merge(Zoom)
