# Supplementary material for: Unsupervised clustering reveals noncanonical myeloid cell subsets in the brain tumor microenvironment
Source: Cancer Immunol Immunother. 2025 Jan 3;74(2):63. doi: 10.1007/s00262-024-03920-1 (PMC11699035; doi:10.1007/s00262-024-03920-1)
Supplement: Supplementary file 7 — Supplementary file7 (PDF 1693 KB) [file 262_2024_3920_MOESM7_ESM.pdf]

C

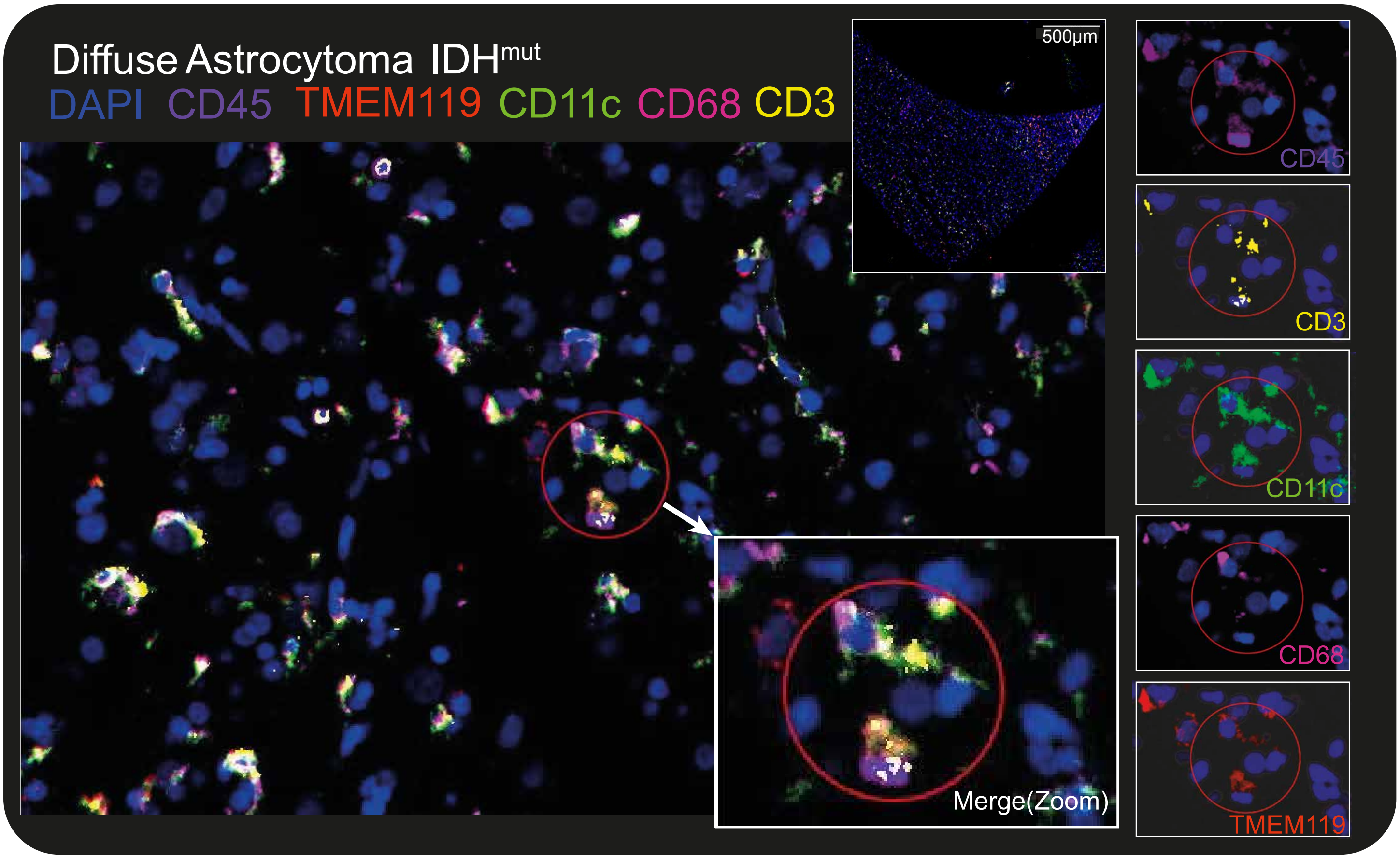
Diffuse Astrocytoma IDHmut
DAPI CD45 TMEM119 CD11c CD68 CD3
500μm
CD45
CD3
CD11c
CD68
TMEM119
Merge(Zoom)

A

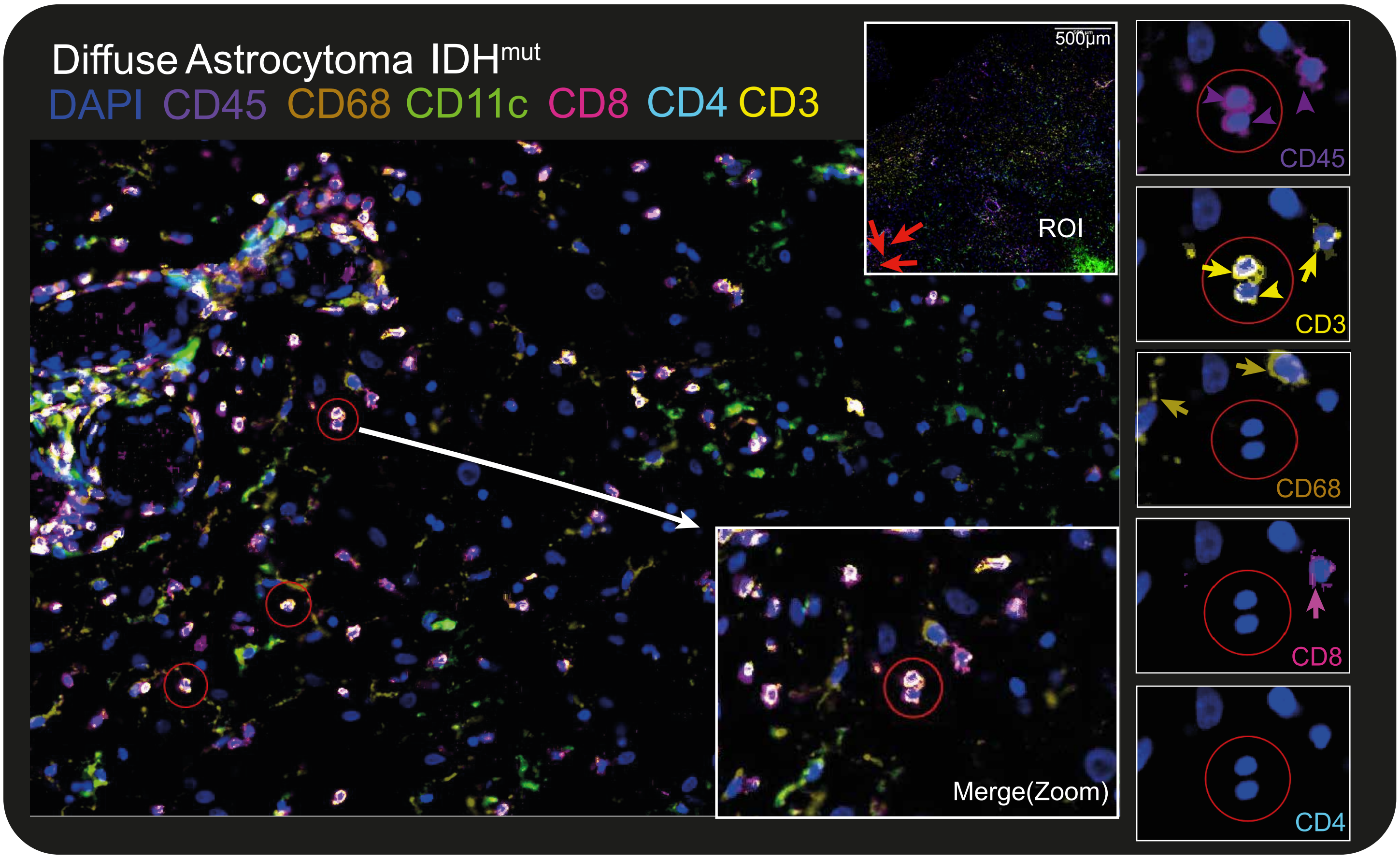
Diffuse Astrocytoma IDHmut
DAPI CD45 CD68 CD11c CD8 CD4 CD3
500μm
ROI
CD45
CD3
CD68
CD8
CD4
Merge(Zoom)
