# Supplementary material for: Unsupervised clustering reveals noncanonical myeloid cell subsets in the brain tumor microenvironment
Source: Cancer Immunol Immunother. 2025 Jan 3;74(2):63. doi: 10.1007/s00262-024-03920-1 (PMC11699035; doi:10.1007/s00262-024-03920-1)
Supplement: Supplementary file 8 — Supplementary file8 (DOCX 12595 KB) [file 262_2024_3920_MOESM8_ESM.docx]

**SUPPLEMENTARY FIGURE LIST**

**Supplementary Figure 1.** **The distribution of phenoclusters based on selected FCM features.** A) Representative flow cytometry (FCM) plots showing the manual gating strategy used for selection of single-events and viability inclusion criteria. B) CD19^+^ myeloid cells show a distinctive CD19 profile (arrow). t-SNE visualization of the fluorescent intensities for the CD19 epitope and the forward scatter height (FSC-H) of viable CD45^+^ cells from all seven brain tumors (as in Fig. 1A). t-SNE, t-distributed stochastic neighbor embedding. C) Separate t-SNE visualizations of phenoclusters. Unsupervised clustering partitioned cells into 25 phenoclusters based on measured surface markers and intrinsic cell properties. t-SNE visualization of CD45^+^ cells colored by phenocluster ID.

Supplementary Fig. 2: **Characteristics of in-house FCM data-related phenoclusters.** A) Immune phenotypes obtained by unsupervised clustering showing phenocluster wise proportions across samples. Columns represent individual samples, and rows represent phenoclusters with annotated phenotypes. Bar plots on the right show cluster event numbers. Despite the influence of sample sizes on phenocluster frequencies, features such as DNT (PC25) in BrMs or abundant microglia in the ODG are conserved; PC, phenocluster; DNT, double-negative T cells; ODG, oligodendroglioma. B) CD4^+^ T cells, macrophages, CD8^+^ T cells, CD3^+^ myeloid cells, neutrophils and CD19^+^ myeloid cells show a median internal cluster stability of over 0.75. Due to instability, DNT (PC3) was not considered further. Boxplot visualization of internal phenocluster stabilities as measured with the Jaccard coefficient based on the assessment of recurrence after multiple and random subsampling cycles (20) of flow cytometry data. Shown in gray, phenoclusters excluded (based on vitality criteria)C) Violin plot visualization of FCM marker FI distributions across phenoclusters. The red line indicates the 95% quantile cutoff from unstained events (universal negative); viability stain indicates cells with poor viability; FSC-A, forward scatter area; SSC-A, side scatter area; FI, fluorescence intensity.

Supplementary Fig. 3: **Markers for distinguishing CNS resident myeloids and the presence of CD3^+^ myeloids in the external mass cytometry data.** Top panel: these results represent the flow cytometry (FCM) dataset from (Friebel et al. 2020)[^9^](https://paperpile.com/c/v9uatz/S5oT) that was analyzed as our in-house FCM data. Discerning infiltrating from resident myeloids in absence of bona fide P2RY12 microglia marker: events expressing P2RY12 generally coexpress CX3CR1 surface marker. A) t-SNE projection of 60,000 viable cells combined from four individuals (far left), color coded for diagnosis; AA, anaplastic astrocytoma; GB, glioblastoma; BrM, brain metastasis. The BrM sample (green) has higher immune cells and lower CD45^-^ counts compared to other samples. The t-SNE projections of individual samples concatenated are shown independently. B) Phenoclusters (far left) obtained by unsupervised clustering of (Friebel et al. 2020) the FCM dataset with PhenoGraph. The CD45 marker FI reveals t-SNE regions enriched for immune cells. t-SNE visualization of FI for the CD45 epitope. CX3CR1^+^ cells coexpressed the bona fide microglial marker P2RY12 (right) and showed low CD45 levels, as expected for microglia; FI, fluorescent intensities; t-SNE, t-distributed stochastic neighbor embedding. Lower panel: analysis of the single-cell mass cytometry (CyTOF) dataset from Friebel et al. (2020) shown in Figure 3. A) CD3^+^ CD14^+^ CD66b^+^ triple-positive cells (arrow, red events) (right) in the proximity of the cells in the CD19^+^ myeloid phenocluster 20 (left) in the UMAP visualization. B) CD3^+^ CD14^+^ CD66b^+^ triple-positive cells (lower arrow, red events) only partly overlap with CD19^+^ CD14^+^ CD66b^+^ triple-positive cells (top arrow, blue events).

Supplementary Fig. 4: **Characterization of** **CD3^+^ myeloid cells with single-cell transcriptome analysis.** A) Overrepresentation analysis (ORA) of the immune gene cluster genes that were differentially expressed between CD3^+^ myeloids vs. all other (noncancer) cells revealed downregulation of genes related to lymphocyte immune response (“positive regulation of lymphocyte activation”) and upregulation of genes related to cellular (“neutrophil chemotaxis”) and humoral immune responses in CD3^+^ myeloids. B) CD3^+^ myeloid downregulation involves cell activation and antigen presentation. Differentially expressed genes between CD3^+^ myeloids versus dendritic cells (DCs, in B)), vs. cytotoxic T cells (T CD8, in C)) or vs. CD4 T cells (in D). Dot size stands for gene counts in the referred pathway, and blue to redshift indicates correlation with adjusted p value.

Supplementary Fig. 5: **Immune cluster genes (ICs) expression patterns across cell types in GB single-cell transcriptome data.** ICs genes in GB single-cell dataset are categorized to appraise overexpressed genes and to estimate the proportion of highly expressed genes within IC subclusters for each cell type (see Methods). A) ICs 6 subcluster 2 genes showing higher expression in myeloid cell types (e.g., CD3^+^ myeloid, macrophage and microglia).

**
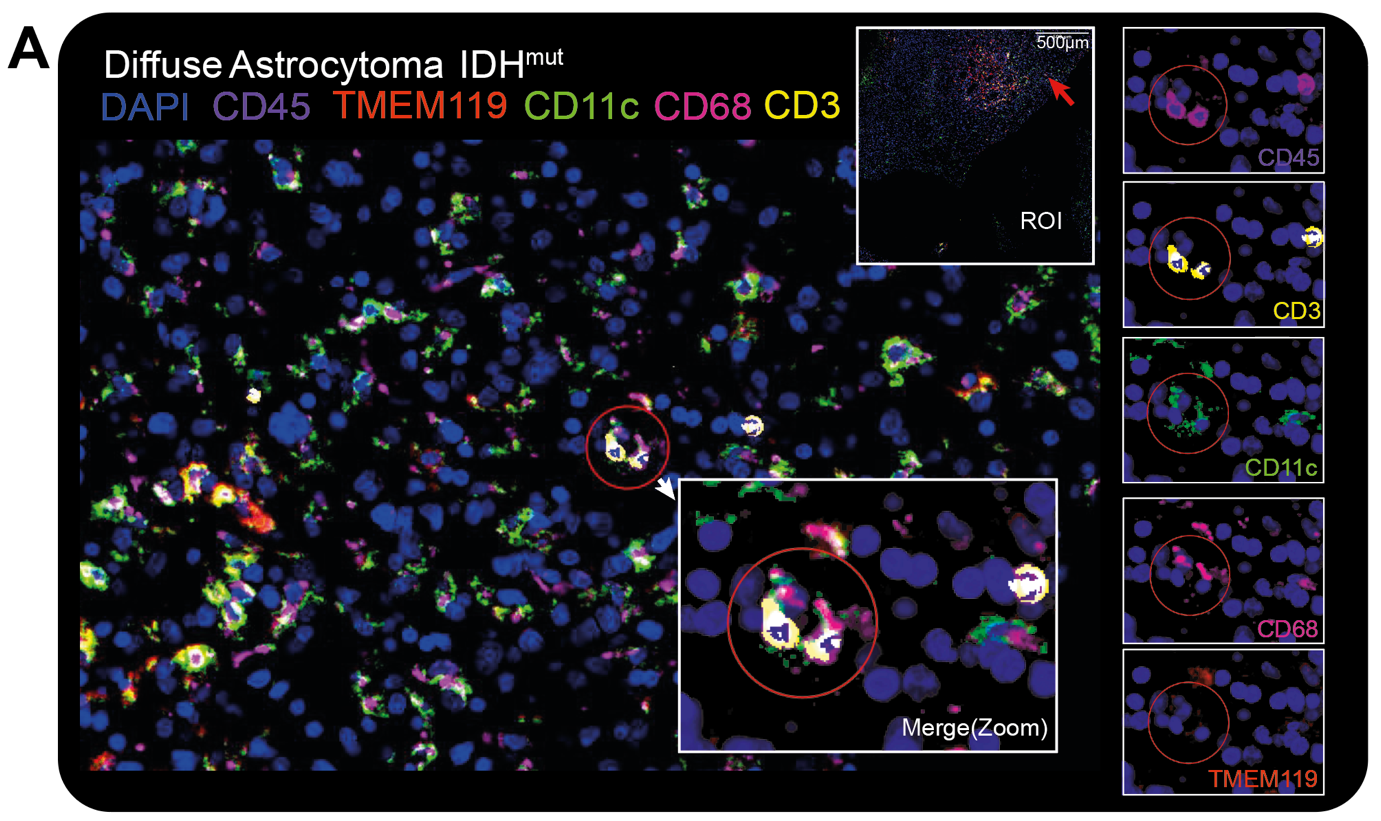
**


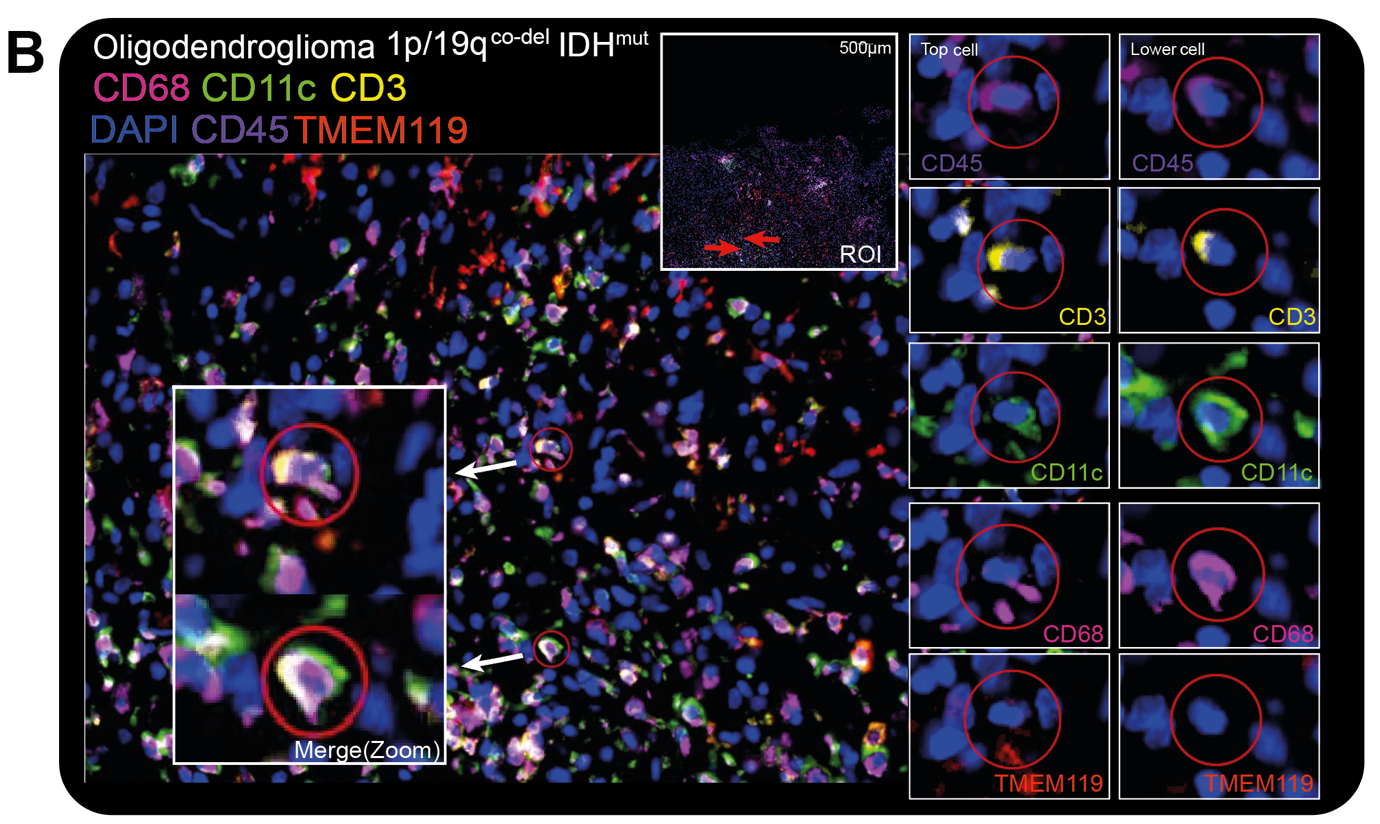


**
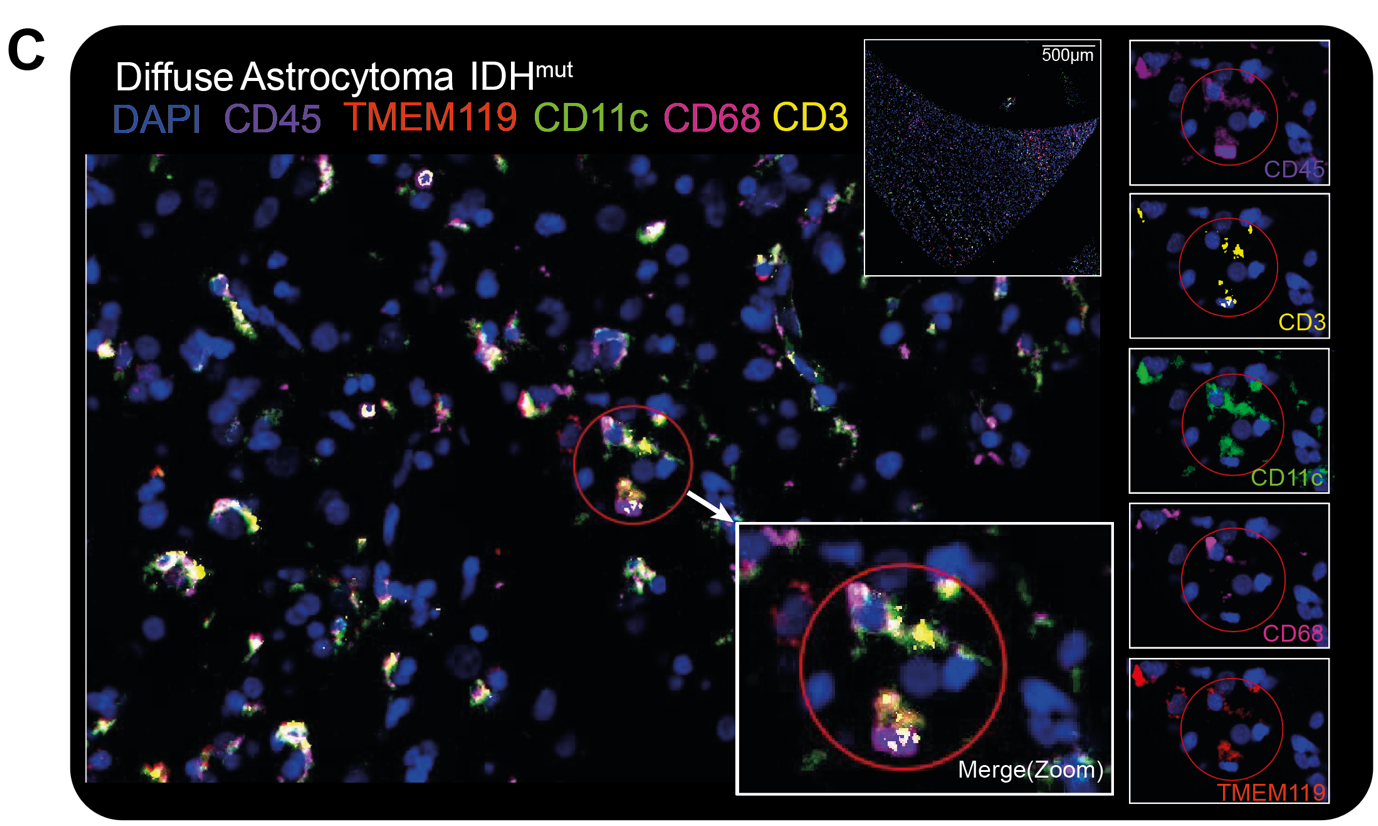
**Supplementary Figure 6. **Evidence of Noncanonical myeloid-like cells from our in-house multiplex-IHC validation cohort.** A-C) Immunostained tissue sections of IDH^mut^ diffuse gliomas with indicated regions of interest (ROIs). C) One borderline case which was still decided to be included. CD3 staining is dotted and co-localizes with TMEM119 in addition to CD45, CD11c, and CD68. Red arrows candidate cell’s location within ROI. The panels on the right shows single stain channels for individual markers. Circles point out noncanonical candidate cells.

**
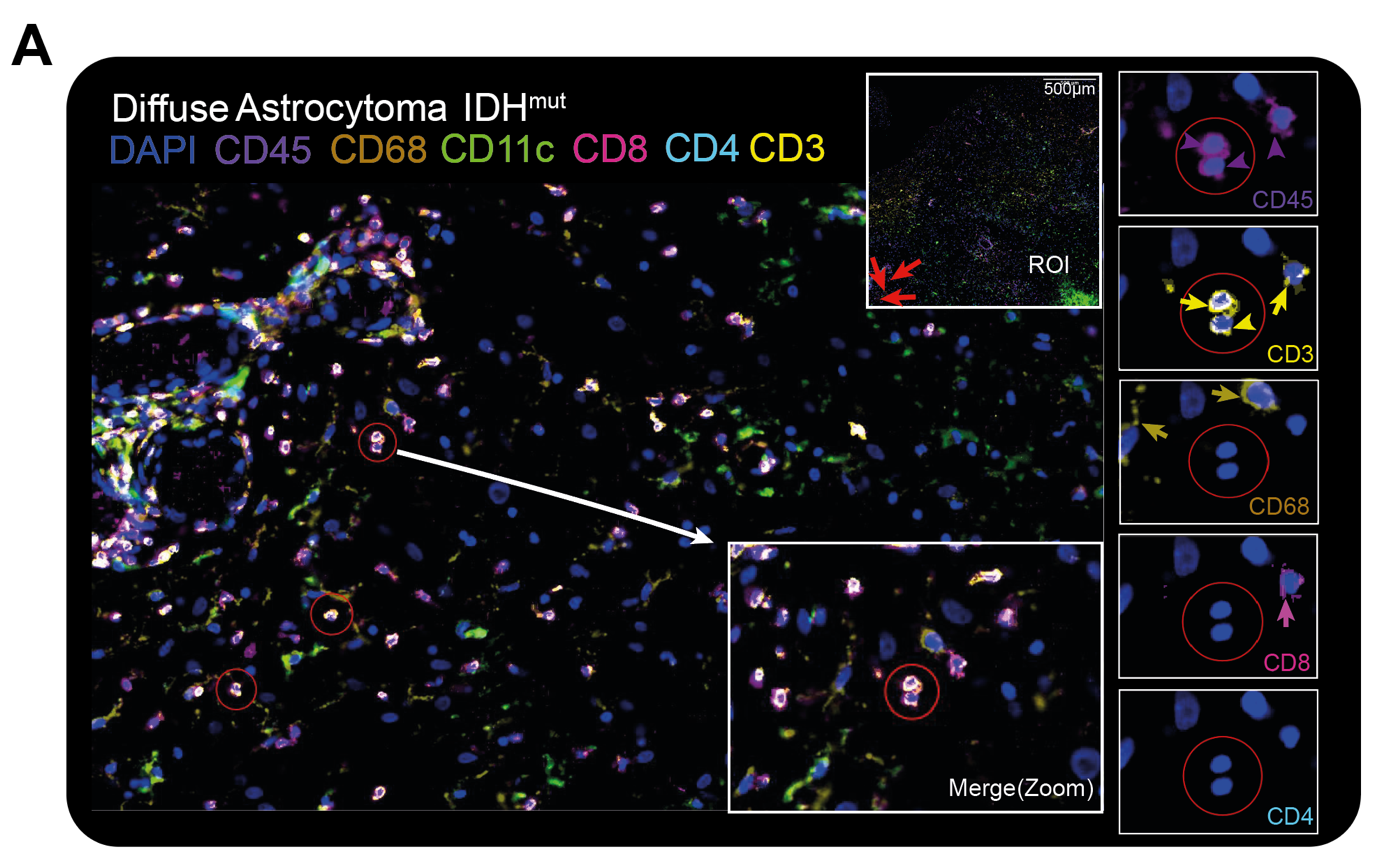
**

Supplementary Figure 7. **Evidence of double negative T lymphocyte (DNT)-like cells from in-house multiplex-IHC validation cohort.** A) Representative image of immunostained tissue sections of IDH^mut^ diffuse gliomas with indicated region of interest (ROIs); red arrows candidate cell’s location within ROI. The panels on the right shows single stain channels for individual markers. Circles point out DNT candidate cells. A CD8^+^ T cell detected on their right side.
