# Supplementary material for: Unsupervised clustering reveals noncanonical myeloid cell subsets in the brain tumor microenvironment
Source: Cancer Immunol Immunother. 2025 Jan 3;74(2):63. doi: 10.1007/s00262-024-03920-1 (PMC11699035; doi:10.1007/s00262-024-03920-1)
Supplement: Supplementary file 13 — Supplementary file13 (DOCX 17 KB) [file 262_2024_3920_MOESM13_ESM.docx]

**Supplementary Table 1**

Related to Figure 1. Clusters generated with PhenoGraph from CD45^+^ cells of in-house FCM and CyTOF datasets. For in-house FCM dataset, based on the FIs of intrinsic cell features (size, FSC-A and granularity, SSC-A), viability stain and cell marker MFIs distributions, cluster phenotypes were annotated (i.e., phenoclusters ID). Thus, immune cells (i.e., phenoclusters) were defined based on the combination of indicated parameters and their relative (FIs) expression. Similarly, accounting for the method and panel differences, CyTOF data was clustered using indicated cell markers (see Methods, CyTOF data processing); FIs, fluorescent intensities; FSC-A, forward scatter area; SSC-A, side scatter area; ID, identity.

**Supplementary Table 2**

Related to Figure 2. Candidate fusion transcripts from RNA-sequencing. Extent of chromosomal rearrangements enquired from number of different gene fusion transcripts at transcriptome level.

**Supplementary Table 3**

Related to Figure 4. Immune subcluster gene lists based on hierarchical clustering.

**Supplementary Table 4**

Related to Supplementary Figure 6 and Supplementary Figure 7. Evidence of noncanonical myeloid-like cells (CD3^+^ myeloids) and double negative T cells (DNTs) from in-house multiplex immunohistochemistry (mIHC) validation cohort, including phenotypes; WHO, World Health Organization; ROI, regions of interest; NA, not available; IDHmut, isocitrate dehydrogenase mutant; IDHwt, isocitrate dehydrogenase wild type.
